# Tailoring Spectral Response and First Hyperpolarizability of Aryl-Substituted BODIPY-Based ‘Push–Pull’ Chromophores: Influence of Medium and Structural Modifications

**DOI:** 10.1021/acs.jpca.5c00383

**Published:** 2025-05-19

**Authors:** Anushree Dutta, Alex Iglesias-Reguant, Josep M. Luis, Ramprasad Misra, Nabanita Deb

**Affiliations:** † School of Chemical Sciences, Indian Association for the Cultivation of Science, Kolkata 700032, India; ‡ Institute of Computational Chemistry and Catalysis and Department of Chemistry, University of Girona, Campus de Montilivi, Girona, Catalonia 17003, Spain; § Institute for Biology, Experimental Biophysics, Humboldt-Universität zu Berlin, Berlin 10115, Germany

## Abstract

The medium plays a pivotal role in dictating the extent of intramolecular charge transfer (ICT) in a molecule, which could be useful in tuning its spectral and nonlinear optical (NLO) response properties. Tuning of ICT in a π-conjugated electronic donor–acceptor molecule has been utilized to modulate the absorption and emission maxima, as well as the first hyperpolarizability (β) of the so-called “push–pull” chromophores. Molecules with boron dipyrromethene (BODIPY)-based acceptors became popular in recent years for their unique photophysical properties, ease of synthesis, and high thermal stability. In this article, we present a quantum chemical investigation of the influence of the medium on the ICT process of some novel aryl-substituted BODIPY molecules. This influence ultimately modulates their absorption, emission, and nonlinear optical (NLO) properties. Both static and frequency-dependent β for the second harmonic generation are investigated along with the Pockels effect. Density functional theory (DFT) and time-dependent DFT (TDDFT) calculations using the long-range corrected CAM-B3LYP functional were employed in the present study. Restricting the rotation of the aryl ring through the incorporation of methyl groups to the BODIPY moiety enhances the fluorescence decay rate of the molecule. Both electronic and vibrational contributions to the static β are considered. A significant increase in β has been observed in polar solvents, compared to that in the gas phase. An interplay between structural and electronic effects was found to dictate the properties investigated. Our results shed light on the ICT process in the studied BODIPY dyes and could be useful in tuning their spectral properties as well as formulating design principles of novel NLO materials for future technological applications.

## Graphical abstract

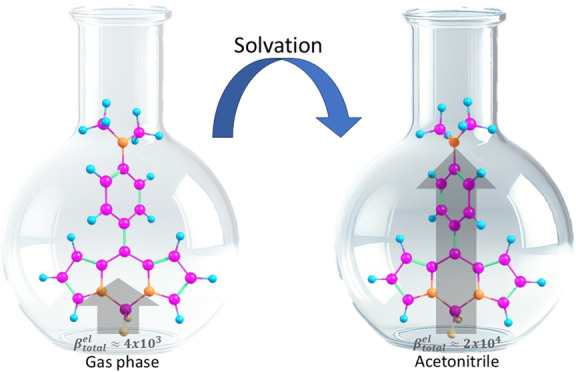

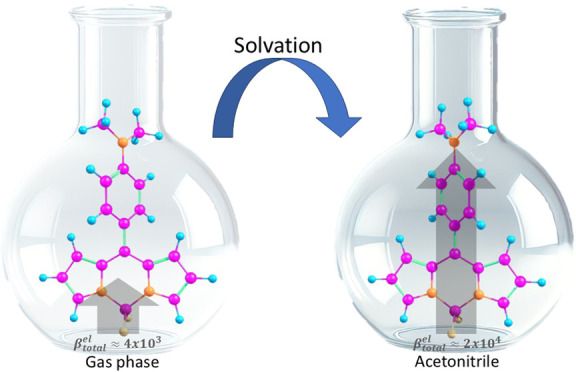

## Introduction

Nonlinear optical (NLO) properties of a material refer to its response to an applied electromagnetic field, in which the relationship between the response and the field intensity is not directly proportional.^1–3^ Owing to its strong NLO characteristics, the material exhibits nonlinear effects such as frequency doubling (second-harmonic generation)^4,5^ frequency mixing (sum and difference frequency generation)^6,7^ optical parametric amplification, among others. NLO materials are highly sought-after due to their potential technological applications in optoelectronic and photonic devices, telecommunications, and optical data storage, among others. Not only have pure organic^8,9^ and inorganic^10^ materials been investigated for their NLO response in recent years, but organic–inorganic hybrid materials^11,12^ have also been explored. Materials based on covalent organic frameworks (COFs) have been reported as promising candidates for future NLO applications.^13^ Of late, advancements in organic NLO response materials have paved the way for the development of next-generation optical devices with improved performance, functionality, and versatility. Organic NLO materials have found applications in various fields, including optical communication, optical switching, frequency conversion, photovoltaics, and biophotonics. Computational methods, such as quantum chemical calculations, have played a crucial role in predicting and optimizing the NLO properties of organic molecules in recent years.^14–20^

Intramolecular charge transfer (ICT)^21^ refers to the redistribution of electron density within a molecule upon electronic excitation, typically from an electron-rich (donor) region to an electron-deficient (acceptor) region. ICT plays a crucial role in determining a material’s nonlinear optical properties and photophysical properties.^22^ It lowers the energy gap between the ground and excited states, leading to red-shifted absorption and emission, and enhances the first hyperpolarizability. The alignment of ICT along the molecular dipole moment enhances the anisotropy of the nonlinear response. The solvent polarity can significantly affect the ICT process in aryl-substituted BODIPYs. In more polar solvents, the charge separation during ICT is better stabilized. This makes these dyes sensitive to environmental changes, a feature useful for solvatochromic sensing. Asymmetric molecules with strong ICT exhibit high first hyperpolarizability, enhancing their NLO response and enabling effects like second-harmonic generation. Thus, ICT-active molecules are crucial for the design of new NLO materials and devices.^22–24^ Organic molecules containing a donor and acceptor group connected via a π-bridge, often referred to as “push–pull” systems, are ideal for ICT and have become increasingly popular for designing new molecules with large NLO properties. Optimizing the donor/acceptor groups and the π-bridge is crucial for achieving effective ICT in “push–pull” molecules, which enhances their NLO properties. BODIPY dyes (4,4-difluoro-4-bora-3a,4a-diaza-s-indacene) are highly sought after as NLO active materials due to their versatile solubility in various solvents, intense absorption, strong emission, and high fluorescence quantum yield. The synthesis of BODIPY dyes was first reported by Treibs and Kreuzer in 1968.^25^ These dyes have since found extensive applications as chemosensors and fluorosensors in biochemical labeling, light-emitting devices, solar cells, pulsed dye lasers, and more.^26^ Sekar and colleagues have demonstrated the charge transfer characteristics in BODIPY-benzimidazole conjugate dyes using UV–vis spectroscopy, steady-state, and time-resolved fluorescence spectroscopy.^27^ Poddar et al. reported the recent advances of BODIPY-based π-conjugated derivatives for their potential applications in photonic and electronic devices.^28^ To fully harness the potential of BODIPY dyes in designing NLO chromophores, a comprehensive understanding of the factors influencing their ICT process is essential.

In this paper, we evaluate the influence of the medium on ICT, and consequently, the spectral response and the linear and nonlinear properties of some novel aryl-substituted “push–pull” BODIPY chromophores. We have computed the absorption, emission, and electronic and vibrational contributions to static and dynamic first hyperpolarizability of these molecules in the gas phase, as well as in cyclohexane, acetonitrile, and water. We have also investigated how the substitution of donor/acceptor groups, changes in properties of the medium, and restriction of rotation of the aryl group through substitution of the methyl group affect the fluorescence decay time in the studied molecules. The polarity of the medium significantly affects the ICT process, and an interplay of structural and charge transfer processes was found to dictate the absorption, emission, and NLO response properties of the studied molecules.

## Methods

The ground state geometries of BODIPY molecules I–VI (see Scheme 1 and Table 1) have been optimized using density functional theory with the CAM-B3LYP functional and the cc-pVTZ basis set. The choice of an appropriate functional and basis set combination is reported to be crucial in studying the properties of a molecule.^29^ CAM-B3LYP, a Coulomb-attenuated hybrid exchange-correlation functional, is widely used for computing the properties of charge-transfer-based molecules.^30^ A previous study^31^ demonstrated that the use of CAM-B3LYP functional in conjunction with 6–31G­(d,p), 6–31+G­(d,p), and cc-pVTZ basis set produced reliable trends in the nonlinear optical response of aryl-substituted BODIPY dyes. Therefore, in the present study, the intramolecular charge transfer characteristics and NLO properties are analyzed using this functional and basis set combination. Solvent effects are accounted for in all calculations using the polarizable continuum model^32^ (PCM) in the framework of self-consistent reaction field (SCRF) theory.

**Scheme 1:**
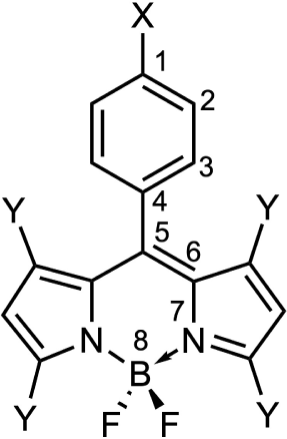
General Structure and Atom Numbering (for Reference) of the Aryl-Substituted BODIPY Molecules Analyzed in this Study

**Table 1:** Substituents and Molecule Labeling of the Aryl-Substituted BODIPY Molecules Shown in Scheme 1

	Substituents	
Compound	X	Y
I	H	CH_3_
II	H	H
III	N(CH_3_)_2_	CH_3_
IV	N(CH_3_)_2_	H
V	NO_2_	CH_3_
VI	NO_2_	H

The optimized structures were confirmed through Hessian evaluation to ensure the absence of imaginary frequencies, verifying that either true local or global minima were obtained on the potential energy surface. All calculations were performed using the Gaussian 16 ^33^ computational chemistry package, and visualizations were generated using GaussView 6.0 ^34^ graphical interface software. Based on the optimized structures, time-dependent density functional theory (TDDFT) calculations were carried out for the first 10 excited states of the molecules. This was followed by calculations of molecular properties related to linear and nonlinear optical behavior.

We calculated the dipole moment, μ, of a molecule from its components as defined below.

1 $$\mu =\sqrt{\mu_{x}^{2}+\mu_{y}^{2}+\mu_{z}^{2}}$$

The average linear polarizability,^1^ α, of the studied molecules was calculated using the expression:

2 $$\alpha =\frac{\alpha_{xx}+\alpha_{yy}+\alpha_{zz}}{3}$$

To analyze the nonlinear optical properties, we computed the first hyperpolarizability, β, which quantifies a molecule’s ability to interact nonlinearly with electric fields, enabling optical phenomena such as second-harmonic generation (SHG) and electro-optic modulation. To provide a comprehensive description of β, we calculated both the total hyperpolarizability, β_total_, which reflects the overall nonlinear response of the system, and the vector component, β_vec_, which gives insight into the directionality of the response relative to the applied electric field. While the general second-rank tensor first hyperpolarizability, β_*ijk*_, has 27 independent components, this number may be reduced due to molecular symmetry. In the static limit, where Kleinman symmetry applies, the additional permutations β_*ijk*_ = β_*ikj*_ = β_*jik*_ = β_*jki*_ = β_*kij*_ = β*_kji_* further reduce the number of unique components of the first hyperpolarizability tensor to 10. In this case, the total hyperpolarizability, β_total_, can be defined as

3 $$\beta_{\mathrm{total}}=\sqrt{\underset{i=x,y,z}{\sum }{\left(\underset{j=x,y,z}{\sum }\beta_{ijj}\right)}^{2}}$$

while the β_vec_ may be described as

4 $$\beta_{\mathrm{vec}}=\frac{\underset{i=x,y,z}{\sum }\left(\underset{j=x,y,z}{\sum }\beta_{ijj}\times \mu_{i}\right)}{\mu }$$

However, these equations are only valid for static cases. For a general dynamic first hyperpolarizability, the reduction of the independent components due to Kleinman symmetry is no longer applicable due to the time-dependent nature of the response. As a result, the reduction of the components β_*ijj*_, β_*jij*_, and β_*jji*_ into β_*ijj*_ is no longer valid. Therefore, β_total_ is expressed as

5 $$\beta_{\mathrm{total}}=\sqrt{\underset{i=x,y,z}{\sum }{\left(\frac{\sum_{j=x,y,z}\left(\beta_{ijj}+\beta_{jij}+\beta_{jji}\right)}{3}\right)}^{2}}$$

and β_vec_ may be defined as^35^

6 $$\beta_{\mathrm{vec}}=\frac{1}{3}\frac{\underset{i,j=x,y,z}{\sum }\left[\left(\beta_{ijj}+\beta_{jij}+\beta_{jji}\right)\times \mu_{i}\right]}{\mu }$$

Frequency-dependent first hyperpolarizabilities (dynamic first hyperpolarizabilities) for the second harmonic generation (β­(−2ω;ω,ω)) and the Pockels effect (β­(−ω;ω,0)) were computed for molecules I–VI in the gas phase, cyclohexane, acetonitrile and water at 1064 nm (0.0428 hartree) using Gaussian 16.^33^

The ratio between $\beta_{\mathrm{vec}}^{el}$ and $\beta_{\mathrm{total}}^{el}$ yields significant insights into the direction of charge transfer within the molecules:^36^

7 $$\beta_{\mathrm{vec}}^{el}/\beta_{\mathrm{total}}^{el}=\cos\theta$$

where θ represents the angle between the dipole moment vector and $\beta_{\mathrm{vec}}^{el}$ components.

Nonlinear optical properties such as β arise from electronic (β^*el*^), nuclear relaxation (β^*nr*^), and curvature (β^*curv*^) contributions. Whereas the electronic component describes the pure electronic response to an external electric field at its equilibrium geometry, the nuclear relaxation and curvature contributions arise from the change of the electronic and vibrational energies due to the field-induced change of the equilibrium geometry and the shape of the potential energy surface, respectively.^37^ Curvature contributions are computationally far more expensive and usually smaller than the other contributions. Accordingly, we define in this study the total first hyperpolarizability (β^*el*+*nr*^) as

8 $$\beta^{el+nr}=\beta^{el}+\beta^{nr}$$

While calculating the electronic contribution is computationally feasible for small and medium-sized molecules using accurate ab initio methods, obtaining the nuclear relaxation contribution is computationally more demanding. It requires either field-dependent optimizations maintaining the Eckart conditions^38,39^ or the calculation of the derivatives of the Hessian, dipole moment, and polarizability with respect to the nuclear coordinates.^37,40^ Both approaches are computationally more expensive than the calculation of the electronic contribution. Consequently, it is common to compute only the electronic contribution. However, recent studies have emphasized the importance of the nuclear relaxation contribution to α and β in understanding the overall properties of molecules and molecular complexes.^41–44^

The infinite optical frequency (IOF) approximation provides an efficient method for calculating dynamic vibrational hyperpolarizabilities with satisfactory accuracy for visible and UV radiation.^40,45^ The IOF approximation assumes that the optical frequency approaches the infinite limit (ω → ∞), which simplifies the expression for the nuclear relaxation contributions to Pockels’ first hyperpolarizability (β^*nr*^(−ω;ω,0)_ω → ∞_).

The field-induced vibrational coordinates (FIC) methodology^46,47^ has been employed for the analytical calculation of β^*nr*^(0;0,0) and β^*nr*^(−ω;ω,0)_ω → ∞_. FICs are defined as the displacement coordinates derived from the change in the equilibrium geometry induced by a static applied field. This approach significantly reduces the number of *n*-th order derivatives with respect to the vibrational coordinates required to compute the nuclear relaxation hyperpolarizabilities. For example, to evaluate the complete tensor of β^*nr*^(0;0,0) of the molecules studied here, using normal modes coordinates requires calculating approximately 4.5 × 10^4^ third derivatives of the energy with respect to vibrational coordinates. In contrast, using FICs, the analytical formulas involve only 10 third derivatives.^46,47^

The radiative decay rates of BODIPY molecules (I–VI) were calculated using FCClasses 3.0.3 to investigate the influence of substituents on the spectral properties of the BODIPY moiety.^48^ The evaluation process involves three main steps: (i) geometry optimization and frequency calculation for the two relevant electronic states, (ii) selection of the appropriate vibronic model, and (iii) computation of the decay rates. The decay rate is obtained using the time-dependent (TD) method by evaluating the integral involving the Fourier transform of the autocorrelation function of the dipole moment operator. According to Fermi’s golden rule^49^ the transition rate between two electronic states^50–52^ is given by

9 Kif=(2π/ℏ)|<ψf|H′^|ψi>|2ρ(Ef)

where, ρ­(*E*_*f*_) is the density of the final state and <ψf|H′^|ψi> is the perturbation matrix between the states. We use the dipole moment operator as a perturbation operator to calculate the fluorescence decay rate.

The Adiabatic Hessian (AH) model is typically employed when the initial and final electronic states exhibit minimal geometric differences. However, in cases where there is significant structural change, such as the large rotation of the *NO*_2_ group from the excited to the ground state, the AH model may become unreliable. In such cases, the Vertical Hessian (VH) or Vertical Gradient (VG) models often provide more robust alternatives.^53,54^ The VG model is a simplified version of the VH model. While the VH approach requires computation of the Hessian for the excited state, the VG model avoids this by using only the gradient at the excited-state optimized geometry. In the VG model, the same set of normal modes and vibrational frequencies is used for both the initial and final states. The displacement is estimated using the gradient of the excited state, making the VG method significantly more computationally efficient. Internal coordinates are employed in the VG model to enhance numerical stability and accuracy.

Factors such as the broadening function and the choice of coordinate system (Cartesian or internal) also influence the decay rate. The homogeneous broadening due to the finite lifetime of the vibrational states, represented by a Lorentzian line shape (HWHM = 0.01), is influenced by the Heisenberg uncertainty principle. While Lorentzian broadening has a negligible effect on the radiative decay rate, it significantly impacts the nonradiative rate by enabling transitions between vibrational states with large energy mismatches, thus increasing the internal conversion rate.

## Results and Discussion

### Electronic Structure of the Studied Molecules in the Gas Phase, Cyclohexane, Acetonitrile, and Water

The electronic structure of the studied molecules was optimized as described in the previous section. Figure 1 depicts the optimized structures of molecules I–VI in acetonitrile, while Figures S1–S3 present the optimized structures of molecules I–VI in the gas phase, water, and cyclohexane, respectively. Table S1 summarizes the key bond lengths from the optimized structures in the gas phase, cyclohexane, acetonitrile, and water. The medium exerts a negligible influence on the geometry optimization, with bond lengths differing by no more than the third decimal place.

**Figure 1:**
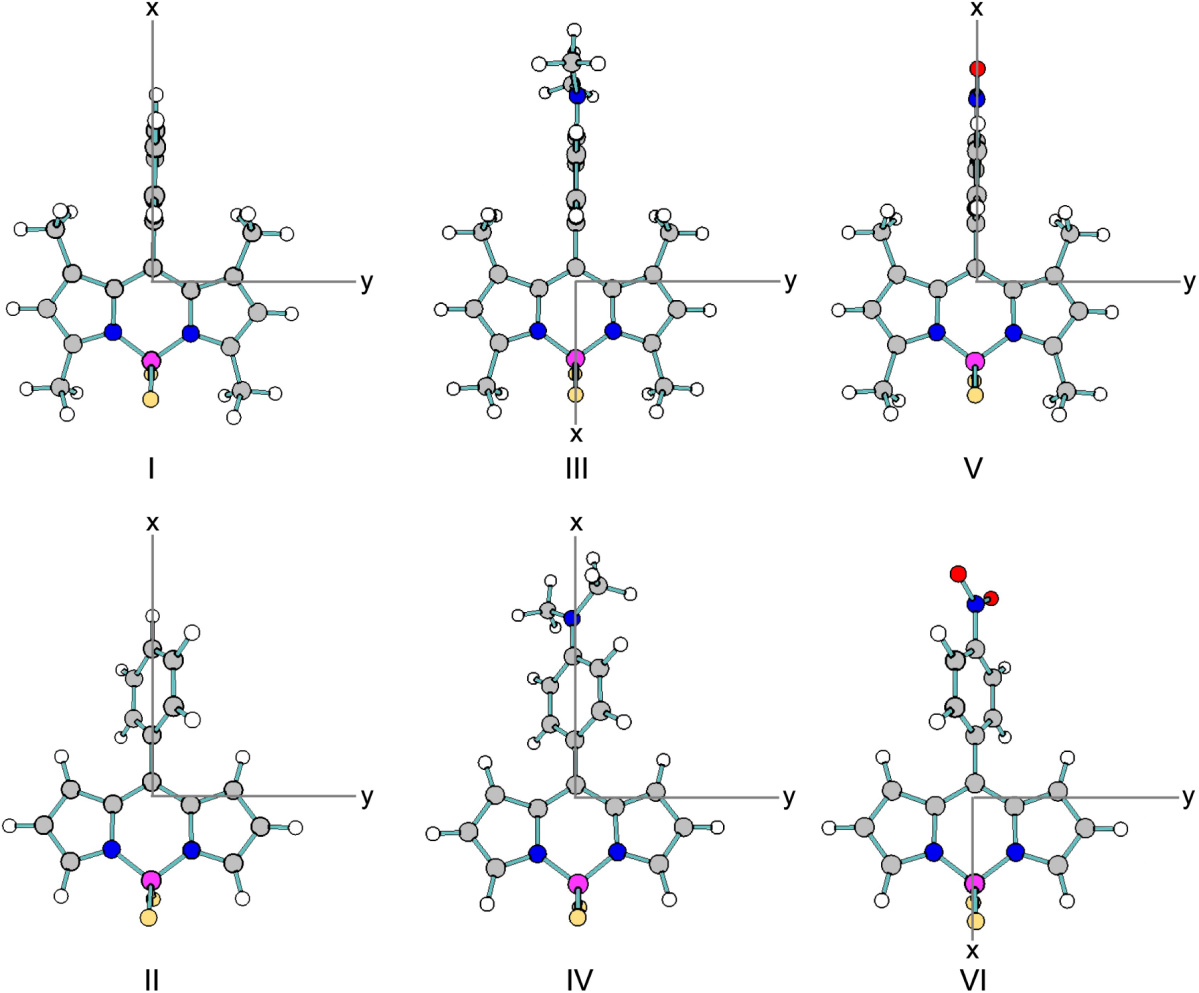
Optimized geometries of aryl substituted BODIPY molecules I–VI in acetonitrile, calculated at the CAM-B3LYP/cc-pVTZ level of theory.

The substitution of donor or acceptor groups on the aryl group leads to noticeable changes in bond lengths. Specifically, the replacement of H or NO_2_ substituents with an NMe_2_ substituent induces significant variations in certain bond distances. However, these changes remain relatively small in the context of the overall molecular structure.

The addition of a methyl group to the BODIPY moiety significantly alters the dihedral angle between the aryl group and the BODIPY core (atoms 3–4–5–6, Scheme 1). The molecules with methyl group substituents (I, III, and V) exhibit a perpendicular dihedral angle (Table 2). In contrast, molecules II, IV, and VI display dihedral angles of 56^◦^, 46^◦^, and 59^◦^, respectively, in acetonitrile. This trend is consistent across the gas phase, cyclohexane, and water as well.

**Table 2:** Dihedral Angle (3-4-5-6, Scheme 1) in Degrees of Aryl-Substituted BODIPY Molecules I–VI in the Gas Phase, Cyclohexane, Acetonitrile, and Water, Respectively

Solvent	I	II	III	IV	V	VI
Gas phase	90.00	57.33	90.18	50.38	90.00	59.52
Cyclohexane	90.00	56.87	90.51	49.00	90.00	59.82
Acetonitrile	90.00	55.67	90.21	46.55	90.00	59.21
Water	90.00	55.58	92.50	46.42	90.00	59.15

### Frontiers Molecular Orbitals and Band Gaps

The gap between the highest occupied molecular orbital (HOMO) and the lowest unoccupied molecular orbital (LUMO) plays a crucial role in the ICT process, consequently affecting the NLO properties of the molecules.^55^ The HOMO and LUMO determine the electron-donating and electron-accepting abilities of a molecule, respectively. The energies of HOMO, LUMO, and the energy gap between them for all molecules (I–VI) are detailed in Table 3, while the frontier molecular orbital depictions in acetonitrile are presented in Figure 2. Generally, the hyperpolarizability of a molecule increases as the HOMO–LUMO gap decreases.^56,57^ However, several other factors also influence the hyperpolarizability. The analysis of these results can be approached from two perspectives: based on the donor/acceptor substitution group or the methyl substitution group.

**Table 3:** Energies of Frontier Molecular Orbitals (HOMO and LUMO) in eV and HOMO-LUMO Gap Δ*E* in eV of the Aryl-Substituted BODIPY Dyes at the CAM-B3LYP/cc-pVTZ Level of Theory

Molecule	Solvents	HOMO	LUMO	Δ*E* (eV)
I	Gas phase	–6.77	–1.55	5.22
	Cyclohexane	–6.83	–1.60	5.23
	Acetonitrile	–6.95	–1.71	5.24
	Water	–6.96	–1.71	5.24
II	Gas phase	–7.38	–2.06	5.31
	Cyclohexane	–7.40	–2.07	5.32
	Acetonitrile	–7.48	–2.14	5.34
	Water	–7.49	–2.14	5.34
III	Gas phase	–6.61	–1.38	5.23
	Cyclohexane	–6.72	–1.48	5.24
	Acetonitrile	–6.84	–1.65	5.19
	Water	–6.84	–1.65	5.18
IV	Gas phase	–7.13	–1.89	5.24
	Cyclohexane	–7.02	–1.88	5.14
	Acetonitrile	–6.92	–2.01	4.90
	Water	–6.91	–2.02	4.89
V	Gas phase	–7.05	–1.86	5.18
	Cyclohexane	–7.02	–1.82	5.19
	Acetonitrile	–7.02	–1.81	5.21
	Water	–7.03	–1.81	5.21
VI	Gas phase	–7.67	–2.48	5.19
	Cyclohexane	–7.60	–2.39	5.21
	Acetonitrile	–7.55	–2.33	5.22
	Water	–7.55	–2.33	5.22

**Figure 2:**
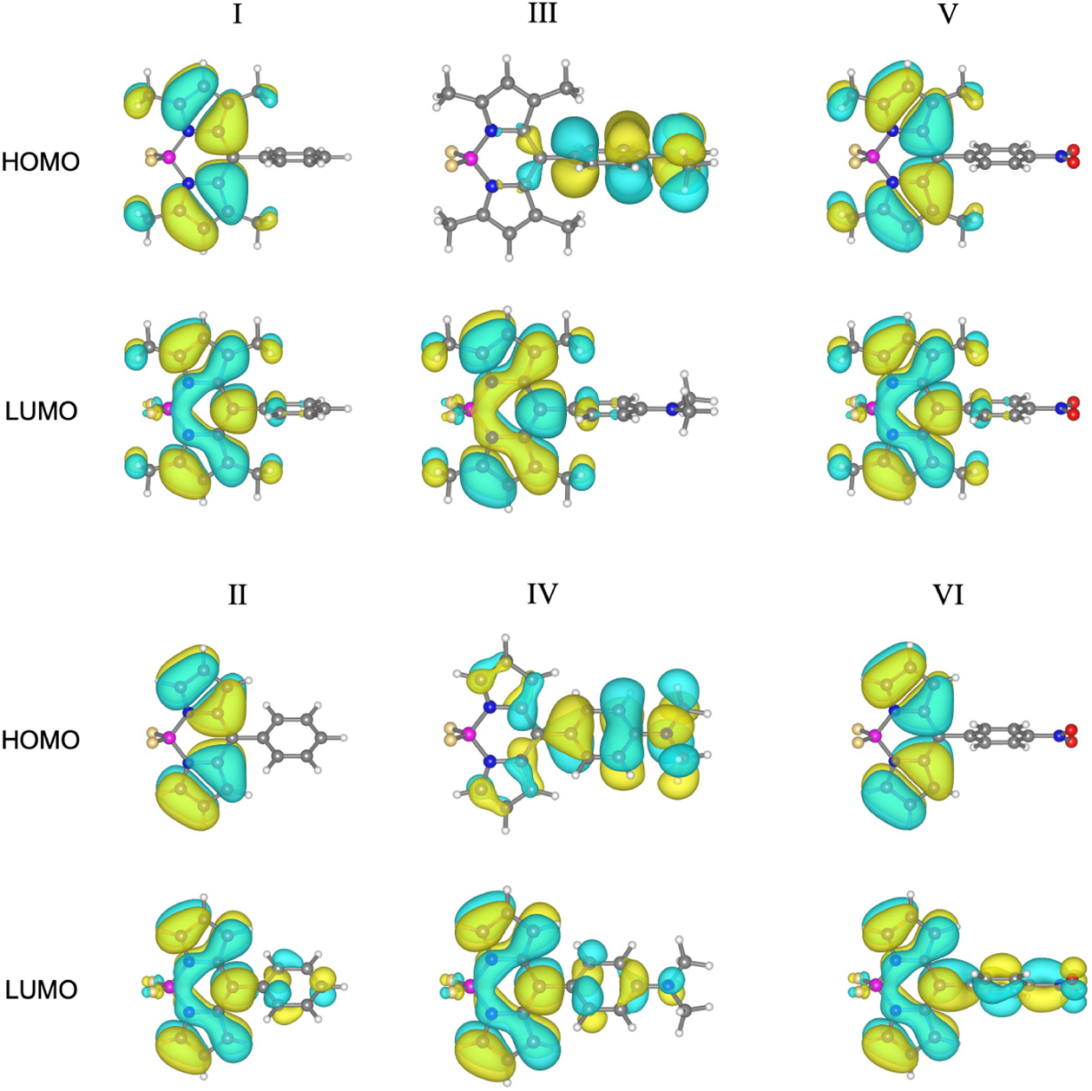
Frontier molecular orbitals of molecules I–VI in acetonitrile at the CAM-B3LYP/cc-pVTZ level of theory.

A comparison between the energy gaps of molecules I, III, and V reveals that the substitution of the donor/acceptor group has a minimal effect in the gas phase and cyclohexane. In contrast, in acetonitrile and water, a significant decrease in the HOMO–LUMO gap is observed when comparing molecules II and VI to molecule IV.^22^ Furthermore, the change in the HOMO–LUMO gap between methyl-substituted and methyl-unsubstituted pairs of molecules (I and II, III and IV, and V and VI) is generally small. However, a significant decrease in the HOMO–LUMO gap is observed when transitioning from molecule III (methyl-substituted) to molecule IV (unsubstituted) in both acetonitrile and water. When analyzing the effect of the medium on the energy gap, it is observed that the use of acetonitrile and water generally increases the energy gap for molecules I, II, V, and VI, while it decreases the gap for molecules III and IV. On the other hand, for all molecules except III, the energy gap in cyclohexane lies between the values observed in the gas phase and those in polar solvents. Although these changes are generally small, the solvent effect on the energy gap of molecule IV is particularly notable. In conclusion, molecule II possesses the highest HOMO–LUMO gap, in contrast to molecule IV, which has the lowest in acetonitrile and water.

In acetonitrile, the molecular orbitals presented in Figure 2 reveal that the electron density in the HOMO is predominantly localized on the BODIPY moiety for molecules containing the hydrogen (H) (I and II) and the electron-withdrawing NO_2_ (V and VI) substituents in the aryl group (see Figures S4–S6 for gas phase, water and cyclohexane molecular orbitals, respectively). In contrast, for molecules with the electron-donating NMe_2_ substituent in the aryl group (III and IV), the electron density in the HOMO is mainly localized on the substituted aryl group. Interestingly, contrary to the HOMO, the electron density in the LUMO is not determined by the aryl-substituted group but by the substitution in the BODIPY moiety. For the methyl-substituted molecules (I, III, and V), the LUMO is localized on the BODIPY moiety. In contrast, for the methyl-unsubstituted molecules (II, IV, and VI), the LUMO is evenly distributed across the entire molecule, indicative of a more delocalized electronic structure compared to the localized pattern observed for their corresponding HOMO. This pattern reflects the molecular substituents’ influence on the electronic structure’s delocalization. The BODIPY core significantly influences the characterization of the HOMO and LUMO in these molecular series, highlighting its crucial role in determining their NLO properties.

### Photophysical Properties

The calculated photophysical properties of molecules I–VI in different media are summarized in Table 4. Both acetonitrile and water induce a similar redshift in the absorption and emission maxima compared to the gas phase, regardless of the specific substituent group. As expected, polar solvents provide greater stabilization of the ground and excited Franck–Condon state energies than nonpolar solvents (Table S2). This stabilization is consistently larger for the excited state than for the ground state across all solvents. However, except for molecule IV, the difference between the stabilization of the excited state (FC) and the ground state is greater in cyclohexane than in polar solvents. This differential stabilization leads to a redshift of the absorption maximum in cyclohexane compared to polar solvents. Conversely, at the excited-state minimum-typically associated with a higher dipole moment-polar solvents offer enhanced stabilization through solute–solvent interactions. As a result, the fluorescence maximum is red-shifted in polar solvents relative to nonpolar ones. For absorption, the redshift of the polar solvents with respect to the gas phase is nearly identical across all molecules, except for molecule IV, where the effect of adding a polar solvent is significantly more pronounced. A comparison of molecules I, III, and V shows that changes in the substituent on the aryl group have minimal impact on the absorption maximum. While the differences among molecules II and VI are slightly larger, they remain relatively modest. The only clear exception to this general trend is molecule IV in a polar solvent. In general, the addition of the electron-donating NMe_2_ group leads to a small absorption maximum blueshift, whereas introducing the electron-withdrawing NO_2_ group on the aryl ring induces a slight redshift. Regarding the effect of methyl substitution in the BODIPY core, a blueshift is observed for the methyl-unsubstituted molecules compared to their methyl-substituted analogs, except for molecule IV in polar solvents.

**Table 4:** Summary of the Electronic Structure Calculations of Molecules I–VI in Gas Phase, Cyclohexane, Acetonitrile, and Water Using TD-DFT at CAM-B3LYP/cc-pVTZ Level of Theory^a^

Molecule	Solvents	λ_abs_	*f* _abs_	Dom. trans.	λ_emi_	*f*_emi_	Dom. trans.	Stokes shift (*cm* ^–1^)
I	Gas phase	410	0.534	H-L	425	0.514	H-L	861
	Cyclohexane	430	0.660	H-L	445	0.642	H-L	784
	Acetonitrile	426	0.632	H-L	483	0.841	H-L	2770
	Water	425	0.629	H-L	484	0.850	H-L	2868
II	Gas phase	398	0.441	H-L	420	0.371	H-L	1316
	Cyclohexane	418	0.557	H-L	434	0.507	H-L	882
	Acetonitrile	413	0.525	H-L	468	0.704	H-L	2846
	Water	413	0.522	H-L	470	0.713	H-L	2936
III	Gas phase	409	0.525	H-L	423	0.508	H-L	809
	Cyclohexane	429	0.649	H-L	443	0.635	H-L	737
	Acetonitrile	425	0.622	(H-1)-L	481	0.837	H-L	2739
	Water	424	0.619	(H-1)-L	483	0.846	H-L	2881
IV	Gas phase	394	0.423	(H-1)-L	414	0.365	H-L	1226
	Cyclohexane	412	0.532	(H-1)-L	414	0.575	(H-1)-L	117
	Acetonitrile	439	0.481	H-L	509	0.568	H-L	3133
	Water	440	0.482	H-L	512	0.593	H-L	3196
V	Gas phase	413	0.536	H-L	451	0.421	H-L	2040
	Cyclohexane	433	0.661	H-L	467	0.558	H-L	1681
	Acetonitrile	428	0.634	H-L	496	0.793	H-L	3203
	Water	428	0.631	H-L	498	0.804	H-L	3284
VI	Gas phase	403	0.430	H-L	440	0.334	H-L	2087
	Cyclohexane	425	0.548	H-L	453	0.462	H-L	1454
	Acetonitrile	420	0.519	H-L	486	0.668	H-L	3233
	Water	420	0.519	H-L	487	0.668	H-L	3276

a Maximum absorption wavelength (*λ*_abs_, nm), maximum emission wavelength (*λ*_emi_, nm), corresponding oscillator strengths for absorption (*f*_abs_) and emission (*f*_emi_), dominant transition of absorption and emission, and stokes shift (cm^–1^).

The redshift in the emission maximum upon transitioning from the gas phase to acetonitrile and water is between two and four times greater than the corresponding redshift in the absorption maximum for all molecules. A comparison of molecules I, III, and V shows that the introduction of an electron-donating group, such as NMe_2_, results in a slight blueshift of the emission maximum, while the electron-withdrawing NO_2_ group causes a significant redshift compared to the unsubstituted compound. Methyl substitution produces a notable redshift in the emission maximum relative to the hydrogen-substituted molecules. Once again, molecule IV in polar solvents deviates from this general trend.

The Stokes shift, defined as the difference between the emission and absorption maxima (in wavenumbers), is larger in polar solvents than in nonpolar solvents and the gas phase for all molecules. The increased Stokes shifts in polar solvents underscore the role of intramolecular charge transfer (ICT) processes in governing the absorption and emission properties of the BODIPY dyes studied.^22^ The largest Stokes shifts are observed in molecules bearing NO_2_-substituted aryl groups. The introduction of a methyl group has a comparatively smaller impact on the Stokes shift, with the maximum blueshift observed being 455 cm^–1^ in the comparison between molecules I and II in the gas phase. The absorption and emission spectra of molecules I–VI are presented in Figures S7–S9. Table S3 summarizes the vertical excitation maxima, oscillator strengths, and the dominant electronic transitions contributing to these transitions.

The fluorescence decay rates (*k*_*r*_) of molecules I–VI are shown in Table 5. In all cases, the decay rate was found to be lower in the gas phase than in the solvents. The *k*_*r*_ values are highest in polar solvents (acetonitrile and water), lowest in the gas phase, and intermediate in cyclohexane for all molecules. Furthermore, within the same medium, it has been observed that the decay rate is highest for molecules with a methyl group in the BODIPY moiety (I, III and V) compared to those without it (II, IV and VI). These results suggest that the methyl substitution in the BODIPY moiety restricts the movement of the aryl group, which leads to a decrease in the lifetime of the molecule.

**Table 5:** Fluorescence Decay Rate of BODIPY Molecules I–VI in the Gas Phase, Cyclohexane, Acetonitrile, and Water

Molecule	Gas phase	Cyclohexane	Acetonitrile	Water
I	1.905 × 10^8^	2.176 × 10^8^	2.419 × 10^8^	2.426 × 10^8^
II	1.414 × 10^8^	1.793 × 10^8^	2.150 × 10^8^	2.160 × 10^8^
III	1.900 × 10^8^	2.162 × 10^8^	2.420 × 10^8^	2.428 × 10^8^
IV	1.425 × 10^8^	–	1.477 × 10^8^	1.528 × 10^8^
V	1.389 × 10^8^	1.717 × 10^8^	2.156 × 10^8^	2.171 × 10^8^
VI	1.164 × 10^8^	1.506 × 10^8^	1.894 × 10^8^	1.906 × 10^8^

### Linear and Nonlinear Optical Properties

The electronic contributions to dipole moment, μ^*el*^, polarizability, α^*el*^, and overall polarizability, α^*el+nr*^ = α^*el*^ + α^*nr*^, are summarized in Table 6. The nuclear relaxation contribution to the dipole moment is, by definition, always zero. The contribution of the *x* component of the dipole is several orders of magnitude greater than that of the *y* and *z* components in all solvent environments (Table S4). The dipole moments are calculated using eq 1. Except for molecule V, the results clearly show that the polar solvents increase the dipole moment compared to the gas phase in all molecules, with values in cyclohexane falling between the gas phase and polar solvents. A comparison of donor/acceptor-substituted molecules leads to the conclusion that the addition of the electron-withdrawing substituents, such as NO_2_, strongly lowers the dipole moment. On the contrary, adding an electron-donor group, such as NMe_2_, significantly increases the dipole moment relative to the hydrogen-substituted molecule. When comparing methyl-substituted and methyl-unsubstituted molecules, it is observed that methyl-unsubstituted molecules generally have a larger dipole moment than their corresponding counterparts, except for molecule VI in the gas phase. Thus, the dipole moment is highest for molecule IV, regardless of the solvent environment, while it is lowest for molecule VI in the gas phase and molecule V in cyclohexane, acetonitrile, and water.

**Table 6:** Static Electronic Contribution to Dipole Moment (*μ* ^*el*^) in Debye, and the Polarizability (*α* ^*el*^) and Overall Polarizability (*α* ^*el+nr*^) in A.U. for Molecules I–VI in Gas Phase, Cyclohexane, Acetonitrile and Water

Molecule	Medium	μ^*el*^	α^*el*^	α^*el+nr*^
I	Gas phase	4.714	267.30	322.98
	Cyclohexane	5.409	308.49	391.99
	Acetonitrile	6.469	385.68	545.09
	Water	6.517	389.84	552.28
II	Gas phase	5.593	212.92	332.41
	Cyclohexane	6.323	246.81	379.68
	Acetonitrile	7.455	310.31	508.87
	Water	7.507	313.64	493.26
III	Gas phase	7.339	309.28	357.27
	Cyclohexane	8.299	354.34	466.17
	Acetonitrile	9.639	439.22	555.88
	Water	9.700	443.87	647.19
IV	Gas phase	9.235	264.81	448.45
	Cyclohexane	10.616	308.68	482.58
	Acetonitrile	12.865	393.30	626.05
	Water	12.974	397.86	625.12
V	Gas phase	0.487	286.61	378.28
	Cyclohexane	0.230	329.93	491.84
	Acetonitrile	0.339	410.91	650.02
	Water	0.372	415.27	652.05
VI	Gas phase	0.399	231.85	409.02
	Cyclohexane	0.633	267.70	487.31
	Acetonitrile	1.143	334.88	595.46
	Water	1.171	338.41	592.98

The *xx*, *yy*, and *zz* components of the electronic polarizability are summarized in Table S5. The average electronic polarizability has been calculated using eq 2. Like the dipole moment, gas phase calculations yield the lowest values for the electronic and overall polarizability values, regardless of the molecule. On the other hand, adding a methyl group to the BODIPY moiety substantially increases the electronic polarizability. A comparison of donor/acceptor substitution reveals that the electronic polarizability increases upon substitution relative to the unsubstituted counterpart. Moreover, electron-donating groups, such as NMe_2_, result in larger polarizability values compared to electron-withdrawing groups, such as NO_2_.

The last column of Table 6 highlights that, although the electronic contribution to polarizability constitutes the largest portion of the total property, accounting for the nuclear relaxation contribution is essential. Notably, the nuclear relaxation contribution to polarizability ranges from 13% to 45% of the total value, depending on the system (Table S6). The results indicate that methyl-unsubstituted molecules exhibit a larger nuclear relaxation contribution to polarizability. This behavior is expected because the presence of a methyl group on the BODIPY moiety restricts the flexibility of the substituted aryl group, thereby reducing its vibrational motion and consequently the nuclear relaxation contribution. Additionally, the medium exerts a distinct influence on the nuclear relaxation contribution for the two groups. The nuclear relaxation contribution to polarizability is lower in the gas phase than in the solvent. As a result, the total polarizability follows a similar trend to the electronic contribution.

The analysis of the static first hyperpolarizabilities of compounds I–VI reveals a significant dependence on solvent polarity, donor/acceptor, as well as methyl group substitution. The static electronic contribution to the total first hyperpolarizability, $\beta_{\mathrm{total}}^{el}$ , and its vector component, $\beta_{\mathrm{vec}}^{el}$ , along with their corresponding total values incorporating nuclear relaxation, $\beta_{\mathrm{total}}^{el+nr}$ and $\beta_{\mathrm{vec}}^{el+nr}$ , were computed in the gas phase, cyclohexane, acetonitrile, and water. The results are summarized in Tables 7 and S7. The combination of electronic and nuclear relaxation contributions provides a comprehensive understanding of the nonlinear optical behavior of these aryl-substituted BODIPY dyes, which is crucial for their potential applications in optoelectronic devices and molecular photonics.

**Table 7:** Static $\beta_{\mathrm{total}}^{el}$ and $\beta_{\mathrm{total}}^{el+nr}$ of Molecules I–VI in the Gas Phase, Cyclohexane, Acetonitrile, and Water at the CAM-B3LYP/cc-pVTZ Level of Theory

	$$\beta_{\mathrm{total}}^{el}$$			
Molecule	Gas phase	Cyclohexane	Acetonitrile	Water
I	1.13 × 10^3^	1.79 × 10^3^	3.52 × 10^3^	3.63 × 10^3^
II	4.90 × 10^3^	7.90 × 10^3^	1.75 × 10^3^	1.82 × 10^3^
III	6.42 × 10^3^	1.06 × 10^3^	2.33 × 10^3^	2.41 × 10^3^
IV	3.97 × 10^3^	7.38 × 10^3^	1.72 × 10^3^	1.78 × 10^3^
V	1.57 × 10^3^	2.51 × 10^3^	4.73 × 10^3^	4.86 × 10^3^
VI	1.13 × 10^3^	1.89 × 10^3^	3.87 × 10^3^	4.00 × 10^3^

	$$\beta_{\mathrm{total}}^{el+nr}$$			
Molecule	Gas phase	Cyclohexane	Acetonitrile	Water
I	5.45 × 10^3^	3.76 × 10^3^	4.61 × 10^3^	4.17 × 10^3^
II	6.80 × 10^3^	1.52 × 10^3^	3.32 × 10^3^	1.59 × 10^3^
III	9.66 × 10^3^	1.35 × 10^3^	2.13 × 10^3^	2.62 × 10^3^
IV	4.43 × 10^3^	7.38 × 10^3^	1.47 × 10^3^	5.82 × 10^3^
V	1.21 × 10^3^	3.25 × 10^3^	1.06 × 10^3^	7.77 × 10^3^
VI	1.12 × 10^3^	4.99 × 10^3^	3.80 × 10^3^	2.10 × 10^3^

Similar to the dipole moment, the contribution along the *x*-axis toward $\beta_{\mathrm{total}}^{el}$ dominates over the other components. The results indicate that during the polarization process of the investigated molecules, the charge transfer is expected to occur predominantly in the *x*-direction, regardless of the nature of the solvent used. Additionally, for $\beta_{\mathrm{total}}^{el}$ and $\beta_{\mathrm{vec}}^{el}$ , the values increase when using acetonitrile and water, with values in cyclohexane falling in between the gas phase and polar solvent values, as shown in Table 7. For instance, compound I exhibits $\beta_{\mathrm{total}}^{el}$ values of 1.13 × 10^3^, 1.79 × 10^3^, 3.52 × 10^3^, and 3.63 × 10^3^ in the gas phase, cyclohexane, acetonitrile, and water, respectively. It is widely recognized that the electronic first hyperpolarizability tends to increase with decreasing HOMO–LUMO energy gap, although this is not the only factor influencing the property. The value of $\beta_{\mathrm{total}}^{el}$ is highest for molecule IV in polar solvents, which also corresponds to the lowest HOMO–LUMO gap among the studied compounds. When comparing methyl-substituted/unsubstituted pairs (I and II, III and IV, and V and VI), this trend holds. For instance, molecule I has a lower energy gap and larger $\beta_{\mathrm{total}}^{el}$ than molecule II, while molecule III has a larger energy gap and lower $\beta_{\mathrm{total}}^{el}$ than molecule IV, except in the gas phase. Although for molecules V and VI the HOMO–LUMO gap is comparable, molecule V shows larger $\beta_{\mathrm{total}}^{el}$ . However, when accounting for the nuclear relaxation contribution, this rule no longer applies.

The total contribution, including nuclear relaxation, $\beta_{\mathrm{total}}^{el+nr}$ and $\beta_{\mathrm{vec}}^{el+nr}$ (Tables 7 and S7), exhibits significantly larger values due to the inclusion of vibrational effects. For example, compound I shows $\beta_{\mathrm{total}}^{el+nr}$ values of 5.45 × 10^3^ in the gas phase, increasing dramatically to 3.76 × 10^4^, 4.61 × 10^4^, and 4.17 × 10^4^ in cyclohexane, acetonitrile, and water, respectively. However, this trend shows several exceptions, as the nuclear relaxation contribution to the static first hyperpolarizability strongly depends on the anharmonic contributions, which become more significant in molecules with low vibrational frequencies, thereby challenging the assumptions of the harmonic approximation (Table S8). Compounds that feature a nonmethylated BODIPY moiety (II, IV, and VI) exhibit a larger total property than their corresponding methyl-substituted dyes (I, III, and V). Our results highlight the role of nuclear relaxation contribution to the static first hyperpolarizability and suggest that this contribution must be considered when investigating these properties.

The ratio of $\beta_{\mathrm{vec}}^{el}$ / $\beta_{\mathrm{total}}^{el}$ provides information about the direction and extent of charge transfer as shown in eq 7. Interestingly, according to the vectorial components of the electronic hyperpolarizability, the ratio $\beta_{\mathrm{vec}}^{el}$ / $\beta_{\mathrm{total}}^{el}$ is unity for all molecules (Table S9), indicating the directionality of the property along the *x*-axis in this case.

Finally, we have studied the electronic contribution to the frequency-dependent first hyperpolarizabilities for the second harmonic generation (β^*el*^(−2ω;ω,ω)) and the Pockels effect (β^*el*^(−ω;ω,0)) at 1064 nm, which is the wavelength of a commonly used laser, and the nuclear relaxation contribution to Pockels first hyperpolarizability in the infinite frequency approximation (IOF) domain (β^*nr*^(−ω;ω,0)_ω → ∞_) (Tables 8, 9, S10 and S11). The IOF is a very good approximation to compute the nuclear relaxation contribution to the Pockels effect at 1064 nm.^46^ Within the infinite-frequency approximation, β^*nr*^(−2ω;ω,ω)_ω → ∞_ is considered to be zero and is thus assumed negligible at 1064 nm. However, this assumption may not hold in the infrared (IR) frequency region, where nuclear relaxation effects could become significant. All these properties have been calculated using eqs 5 and 6. The dynamic hyperpolarizability results reveal that the first hyperpolarizability is highly frequency-dependent.

**Table 8:** Values of $\beta_{\mathrm{total}}^{el}\left(-\omega ;\omega ,0\right)$ for *ω* = 1064 nm, and $\beta_{\mathrm{total}}^{nr}{\left(-\omega ;\omega ,0\right)}_{\omega \to \infty }$ of Investigated Molecules in Gas Phase, Cyclohexane, Acetonitrile and Water at CAM-B3LYP/cc-pVTZ Level of Theory

	$$\beta_{\mathrm{total}}^{el}\left(-\omega ;\omega ,0\right)$$			
Molecule	Gas phase	Cyclohexane	Acetonitrile	Water
I	1.35 × 10^3^	2.22 × 10^3^	2.54 × 10^3^	2.54 × 10^3^
II	5.95 × 10^3^	1.02 × 10^3^	9.90 × 10^3^	9.79 × 10^3^
III	8.26 × 10^3^	1.43 × 10^3^	1.50 × 10^3^	1.49 × 10^3^
IV	5.32 × 10^3^	1.03 × 10^3^	1.79 × 10^3^	1.83 × 10^3^
V	1.88 × 10^3^	3.07 × 10^3^	3.66 × 10^3^	3.67 × 10^3^
VI	1.38 × 10^3^	2.37 × 10^3^	2.89 × 10^3^	2.90 × 10^3^

	$$\beta_{\mathrm{total}}^{nr}{\left(-\omega ;\omega ,0\right)}_{\omega \to \infty }$$			
Molecule	Gas phase	Cyclohexane	Acetonitrile	Water
I	6.28 × 10^3^	9.92 × 10^3^	2.75 × 10^3^	2.90 × 10^3^
II	2.51 × 10^3^	2.16 × 10^3^	3.64 × 10^3^	3.03 × 10^3^
III	8.40 × 10^3^	1.49 × 10^3^	4.33 × 10^3^	4.72 × 10^3^
IV	4.82 × 10^3^	2.42 × 10^3^	8.78 × 10^3^	9.76 × 10^3^
V	1.20 × 10^3^	1.46 × 10^3^	3.94 × 10^3^	4.18 × 10^3^
VI	2.98 × 10^3^	3.80 × 10^3^	4.65 × 10^3^	4.64 × 10^3^

**Table 9:** Values of $\beta_{\mathrm{total}}^{el}\left(-2\omega ;\omega ,\omega \right)$ for *ω* = 1064 nm of Investigated Molecules in the Gas Phase, Cyclohexane, Acetonitrile and Water at CAM-B3LYP/cc-pVTZ Level of Theory

	$$\beta_{\mathrm{total}}^{el}\left(-2\omega ;\omega ,\omega \right)$$			
Molecule	Gas phase	Cyclohexane	Acetonitrile	Water
I	2.38 × 10^3^	4.49 × 10^3^	3.89 × 10^3^	3.83 × 10^3^
II	1.18 × 10^3^	2.49 × 10^3^	1.71 × 10^3^	1.64 × 10^3^
III	1.78 × 10^3^	3.61 × 10^3^	2.73 × 10^3^	2.64 × 10^3^
IV	1.13 × 10^4^	2.62 × 10^4^	4.83 × 10^4^	4.93 × 10^4^
V	3.15 × 10^3^	5.80 × 10^3^	5.36 × 10^3^	5.30 × 10^3^
VI	2.48 × 10^3^	4.86 × 10^3^	4.45 × 10^3^	4.39 × 10^3^

The calculated β^*el*^(−ω;ω,0) exhibit magnitudes that are generally comparable to static first hyperpolarizability (β^*el*^(0;0,0)) (Table 7). Furthermore, the observed trends regarding substituents are consistent across the systems studied. Similar to $\beta^{el}\left(0;0,0\right)$ , when considering the solvents studied, the property is enhanced, surpassing the gas phase values. For the β^*nr*^(−ω;ω,0)_ω → ∞_, the values are between one and two three of magnitude smaller than the corresponding β^*nr*^(0;0,0) values (Table S7), depending on the role played by the anharmonicity in the static contributions. For instance, the β^*nr*^(−ω;ω,0)_ω → ∞_ for molecules I and II in the gas phase are 6.28 × 10^2^ and 2.51 × 10^3^, respectively, while static β^*nr*^ for the same systems are 4.32 × 10^3^ and 1.67 × 10^5^, respectively. As a general trend, the solvent effects also increase the values of β^*nr*^(−ω;ω,0)_ω → ∞_.

The computed $\beta_{total}^{el}\left(-2\omega ;\omega ,\omega \right)$ (Table 9) reveals that in the gas phase, the values are larger than those of static β^*el*^. This behavior is also observed for cyclohexane. However, under polar solvent environments, the SHG hyperpolarizabilities are very similar to their static β^*el*^ counterparts except for molecule IV. For molecule IV, the β^*el*^(−2ω;ω,ω) value is enhanced by a factor of 4 compared to its static counterpart. Importantly, the trends regarding substituent effects closely mirror those observed for β^el^(0;0,0), with a noticeable attenuation of the trends in the gas phase. Molecule IV exhibits the highest SHG response irrespective of solvent environments (Figure S11).

## Conclusion

Quantum chemical calculations revealed the modulation of the ICT process in novel Aryl-Substituted BODIPY-based “Push–Pull” Chromophores, offering insights for designing novel NLO materials. The effects of medium changes and structural modifications on the absorption, emission, as well as static and frequency-dependent first hyperpolarizabilities have been thoroughly investigated. Long-range corrected DFT functional, CAM-B3LYP, which is suitable for studies of spectral and NLO response properties of ICT molecules, has been employed. Our results demonstrate that the solvent environment plays a crucial role in tuning the spectral properties and first hyperpolarizability of the studied molecules. Specifically, the electronic contribution to the first hyperpolarizabilities is significantly enhanced in polar solvents (acetonitrile, water), compared to the gas phase, with values in a nonpolar solvent (cyclohexane) falling in between. However, the nuclear relaxation contribution might not exhibit the same solvent-dependent trend across all studied systems. Additionally, restricting the rotation of the aryl ring with respect to the BODIPY core through methyl group substitutions leads to an increase in the fluorescence decay rate. This observation suggests a complex interplay between ICT and steric effects induced by the substitutions. The insights gained in this study could provide valuable guidance for the rational design of novel NLO materials with enhanced performance for future technological applications.

## Supplementary Material



## Notes

The optimized structures of Molecules I—VI in the gas phase, cyclohexane, and water; important bond length of optimized molecules; Frontier molecular orbitals of the molecules in the gas phase, cyclohexane, and water; absorption and emission wavelengths and spectra; comparison of dipole moments, polarizability, and first hyperpolarizability in different solvents; coordinates of the optimized geometries of Molecules I—VI in the gas phase, cyclohexane, acetonitrile, and water (PDF)
