# Supplementary material for: Tailoring Spectral Response and First Hyperpolarizability of Aryl-Substituted BODIPY-Based ‘Push–Pull’ Chromophores: Influence of Medium and Structural Modifications
Source: J Phys Chem A. 2025 May 19;129(25):5427–37. doi: 10.1021/acs.jpca.5c00383 (PMC12207575; doi:10.1021/acs.jpca.5c00383)
Supplement: Supplementary file 1 [file jp5c00383_si_001.pdf]

Supporting Information: Tailoring Spectral Response and First Hyperpolarizability of Aryl-Substituted BODIPY-based 'Push-Pull' Chromophores: Influence of Medium and Structural Modifications

Anushree Dutta,[†,§] Alex Iglesias-Reguant,[‡,§] Josep M. Luis,[‡] Ramprasad Misra,[*,¶] and Nabanita Deb[*,†]

†*School of Chemical Sciences, Indian Association for the Cultivation of Science, Kolkata-700032, India*

‡*Institute of Computational Chemistry and Catalysis and Department of Chemistry, University of Girona, Campus de Montilivi, 17003 Girona, Catalonia, Spain*

¶*Institute for Biology, Experimental Biophysics, Humboldt-Universität zu Berlin, 10115 Berlin, Germany*

§*signifies equal contribution*

E-mail: ramprasad.misra@hu-berlin.de; nabanita.deb@iacs.res.in

Figure S1, S2, and S3 are the optimized geometries of molecules I-VI in the gas phase, water, and cyclohexane, respectively. The optimized geometries in acetonitrile are given in the main text. The frontier molecular orbital pictures of molecules I & II, III & IV and V & VI in the gas phase, water and cyclohexane are depicted in Figures S4, S5 and S6 (the frontier molecular orbital pictures in acetonitrile has been illustrated in the main text). The absorption and emission spectra of molecules

I & II, III & IV, and V & VI in the gas phase, cyclohexane, acetonitrile, and water are illustrated in Figures S7, S8 and S9, respectively. The change in dipole moment, polarizability, static and dynamic first hyperpolarizability due to solvation is shown in Figure S10. The Bar plot of Pockels and SHG values of molecules I-VI in gas phase (a) cyclohexane (b) and water (c) is shown in Figure S11. The coordinates of all the optimised geometries of molecules I-VI in the gas phase, cyclohexane, acetonitrile, and water are also provided in Tables S12-S23.

Table S1: Bond length of Ayrl substituted BODIPY molecules I-VI in gas phase, cyclohexane, acetonitrile, and water, respectively.

Molecule	Medium	Bond						
		1-2	2-3	3-4	4-5	5-6	6-7	7-8
I	Gas phase	1.3846	1.3841	1.3876	1.4866	1.3922	1.3932	1.5488
	Cyclohexane	1.3848	1.3843	1.3877	1.4865	1.3922	1.3942	1.5458
	Acetonitrile	1.3852	1.3845	1.3878	1.4864	1.3927	1.3959	1.5406
	Water	1.3853	1.3845	1.3878	1.4864	1.3927	1.3960	1.5404
II	Gas phase	1.3844	1.3829	1.3916	1.4772	1.3935	1.3846	1.5589
	Cyclohexane	1.3847	1.3830	1.3918	1.4766	1.3940	1.3854	1.5537
	Acetonitrile	1.3851	1.3831	1.3925	1.4754	1.3951	1.3868	1.5465
	Water	1.3852	1.3831	1.3925	1.4754	1.3951	1.3868	1.5462
III	Gas phase	1.4028	1.3804	1.3861	1.485	1.3927	1.3933	1.548
	Cyclohexane	1.4038	1.3802	1.3864	1.4846	1.3931	1.3944	1.5450
	Acetonitrile	1.4052	1.3802	1.3868	1.4843	1.3936	1.3961	1.540
	Water	1.4052	1.3802	1.3868	1.4843	1.3938	1.3962	1.5398
IV	Gas phase	1.405	1.376	1.394	1.466	1.398	1.384	1.556
	Cyclohexane	1.4067	1.3754	1.3951	1.4637	1.4006	1.3846	1.5508
	Acetonitrile	1.4095	1.3737	1.3980	1.4581	1.4041	1.3858	1.5430
	Water	1.4096	1.3736	1.3982	1.4578	1.4043	1.3858	1.5426
V	Gas phase	1.380	1.3814	1.3887	1.4864	1.3914	1.3928	1.5499
	Cyclohexane	1.3804	1.3811	1.3888	1.4865	1.3915	1.3938	1.5468
	Acetonitrile	1.3807	1.3808	1.3887	1.4866	1.3917	1.3955	1.5416
	Water	1.3807	1.3808	1.3887	1.4866	1.3916	1.3956	1.5414
VI	Gas phase	1.3796	1.3809	1.3918	1.4793	1.3915	1.3846	1.561
	Cyclohexane	1.3799	1.3807	1.3917	1.4792	1.3915	1.3855	1.5560
	Acetonitrile	1.3803	1.3802	1.3918	1.4786	1.3920	1.3868	1.5485
	Water	1.3803	1.3802	1.3918	1.4786	1.3920	1.3868	1.5482

Table S2: Values of GS and ES (FC) relative energies taking as a reference the gas phase total energies of investigated molecules in cyclohexane, acetonitrile, and water at CAM-B3LYP/cc-pVTZ level of theory.

Molecule	Ground state relative energies (kcal/mol)		
	Cyclohexane	Acetonitrile	Water
I	-2.56	-6.52	-6.70
II	-3.15	-7.66	-7.85
III	-2.99	-7.55	-7.76
IV	-3.85	-9.45	-9.69
V	-3.66	-9.26	-9.51
VI	-4.20	-10.32	-10.58
Molecule	Excited state (FC) relative energies (kcal/mol)		
	Cyclohexane	Acetonitrile	Water
I	-5.75	-9.02	-9.12
II	-6.57	-10.38	-10.49
III	-6.13	-9.99	-10.13
IV	-6.96	-16.86	-17.27
V	-6.93	-11.83	-12.00
VI	-7.67	-13.11	-13.30

Table S3: H and L stands for HOMO and LUMO respectively. (H-1), (H-3), (L+1) and (L+2) represents HOMO-1, HOMO-3, LUMO+1 and LUMO+2 orbitals respectively. The vertical transition maxima (nm) along with corresponding oscillator strengths and dominant transitions for molecules I-VI in the gas phase, cyclohexane, acetonitrile, and water.

Molecule	Medium	Vert. trans. maxima(nm)	Osc. Strength	Dominant trans.
I	Gas phase	410	0.53	H-L(0.69)
	Cyclohexane	430	0.66	H-L(0.70)
	Acetonitrile	426	0.63	H-L(0.69)
	Water	425	0.63	H-L(0.69)
II	Gas phase	398	0.44	H-L(0.69)
	Cyclohexane	418	0.55	H-L(0.69)
	Acetonitrile	413	0.53	H-L(0.69)
	Water	413	0.53	H-L(0.69)
III	Gas phase	236	0.83	(H-1)-(L+2)(0.55)
	Cyclohexane	429	0.64	H-L(0.69)
	Acetonitrile	241	0.98	H-(L+2)(0.57)
	Water	241	0.98	H-(L+2)(0.57)
IV	Gas phase	395	0.42	(H-1)-L(0.69)
	Cyclohexane	412	0.53	(H-1)-L(0.69)
	Acetonitrile	406	0.49	(H-1)-L(0.69)
	Water	405	0.49	(H-1)-L(0.69)
V	Gas phase	412	0.54	H-L (0.69)
	Cyclohexane	433	0.66	H-L(0.70)
	Acetonitrile	251	0.64	(H-3)-(L+1) (0.66)
		428	0.63	H-L (0.69)
	Water	251	0.64	(H-3)-(L+1)(0.66)
		428	0.63	H-L (0.69)
VI	Gas phase	404	0.43	H-L(0.68)
	Cyclohexane	425	0.54	H-L(0.68)
	Acetonitrile	421	0.52	H-L (0.68)
	Water	283	0.52	(H-3)-L(0.64)
		420	0.52	H-L (0.68)

Table S4: x, y and z components of dipole moments (μ^{el}, Debye) for molecules I-VI in gas phase, cyclohexane, acetonitrile, and water employing the CAM-B3LYP/cc-pVTZ level of theory.

Molecule	Solvent	μ_x^{el}	μ_y^{el}	μ_z^{el}	μ^{el}
I	Gas phase	4.714	-0.000	-0.002	4.714
	Cyclohexane	5.409	0.000	-0.003	5.409
	Acetonitrile	6.469	0.001	-0.003	6.469
	Water	6.517	0.001	-0.002	6.517
II	Gas phase	5.593	0.000	-0.002	5.593
	Cyclohexane	6.322	0.010	-0.124	6.323
	Acetonitrile	7.455	0.000	-0.002	7.455
	Water	7.507	0.000	-0.002	7.507
III	Gas phase	7.339	0.107	-0.010	7.339
	Cyclohexane	8.298	0.119	-0.001	8.299
	Acetonitrile	9.638	0.172	-0.007	9.639
	Water	9.698	0.175	-0.009	9.700
IV	Gas phase	9.235	0.008	-0.016	9.235
	Cyclohexane	10.616	0.005	-0.042	10.616
	Acetonitrile	12.865	0.001	-0.011	12.865
	Water	12.974	0.001	-0.009	12.974
V	Gas phase	0.487	0.000	-0.006	0.487
	Cyclohexane	0.230	0.000	-0.004	0.230
	Acetonitrile	0.339	0.000	-0.003	0.339
	Water	0.372	0.000	-0.003	0.372
VI	Gas phase	0.399	0.001	-0.017	0.399
	Cyclohexane	0.633	0.002	-0.018	0.633
	Acetonitrile	1.143	0.001	-0.011	1.143
	Water	1.171	0.001	-0.011	1.171

Table S5: *xx*, *yy* and *zz* components of electronic polarizability (α, a.u) and isotropic averaged α for molecules I-VI in gas phase, cyclohexane, acetonitrile, and water employing the CAM-B3LYP/cc-pVTZ level of theory.

Molecule	Solvent	α_{xx}	α_{yy}	α_{zz}	α
I	Gas phase	157.34	344.02	300.52	267.29
	Cyclohexane	177.11	409.10	339.22	308.47
	Acetonitrile	228.14	529.72	399.04	385.63
	Water	231.58	535.94	401.82	389.78
II	Gas phase	116.33	278.47	243.93	212.91
	Cyclohexane	129.62	333.87	276.91	246.80
	Acetonitrile	160.89	439.71	330.27	310.29
	Water	162.86	445.22	332.77	313.62
III	Gas phase	185.01	365.03	377.77	309.27
	Cyclohexane	208.55	432.55	421.87	354.32
	Acetonitrile	268.33	562.53	486.68	439.18
	Water	272.34	569.50	489.65	443.83
IV	Gas phase	138.16	301.55	354.83	264.85
	Cyclohexane	152.88	357.99	415.40	308.76
	Acetonitrile	188.79	463.36	528.53	393.56
	Water	191.11	468.72	534.58	398.14
V	Gas phase	171.81	349.47	338.54	286.61
	Cyclohexane	194.04	415.52	380.18	329.91
	Acetonitrile	250.58	539.17	442.84	410.86
	Water	254.35	545.61	445.71	415.22
VI	Gas phase	129.37	287.80	278.37	231.85
	Cyclohexane	144.97	345.33	312.79	267.70
	Acetonitrile	181.26	457.32	366.00	334.86
	Water	183.51	463.21	368.43	338.38

Table S6: Nuclear relaxation contribution to polarizability (α^{nr}) in a.u. for molecules I-VI in the gas phase, cyclohexane, acetonitrile, and water.

Molecule	α^{nr}			
	Gas phase	Cyclohexane	Acetonitrile	Water
I	55.68	83.50	159.40	162.44
II	119.49	122.87	198.56	179.62
III	47.99	111.84	116.65	203.32
IV	183.64	173.91	232.75	227.26
V	91.67	161.92	239.11	236.78
VI	177.17	219.61	260.58	254.58

Table S7: Static $\beta^{el}_{\mathrm{vec}}$ and $\beta^{el+nr}_{\mathrm{vec}}$ of molecules I-VI in the gas phase, cyclohexane, acetonitrile, and water at CAM-B3LYP/cc-pVTZ level of theory.

Molecule	$\beta^{el}_{\mathrm{vec}}$			
	Gas phase	Cyclohexane	Acetonitrile	Water
I	-1.13×10^3	-1.79×10^3	-3.52×10^3	-3.63×10^3
II	-4.90×10^2	-7.90×10^2	-1.75×10^3	-1.82×10^3
III	-6.40×10^2	-1.06×10^3	-2.32×10^3	-2.40×10^3
IV	3.97×10^3	7.38×10^3	1.72×10^4	1.78×10^4
V	1.57×10^3	2.51×10^3	-4.73×10^3	-4.86×10^3
VI	-1.13×10^3	-1.89×10^3	-3.87×10^3	-4.00×10^3
Molecule	$\beta^{el+nr}_{\mathrm{vec}}$			
	Gas phase	Cyclohexane	Acetonitrile	Water
I	-5.45×10^3	-1.19×10^4	-3.58×10^4	-3.39×10^4
II	-6.77×10^4	-8.04×10^4	-7.37×10^4	-6.21×10^4
III	2.40×10^3	-8.15×10^3	1.24×10^5	2.04×10^5
IV	3.00×10^5	5.25×10^4	-8.57×10^4	-7.45×10^4
V	1.21×10^4	2.41×10^4	-6.28×10^4	-5.93×10^4
VI	-1.01×10^5	-1.31×10^5	-1.34×10^5	-1.22×10^5

Table S8: Values of $\beta^{nr}_{\texttt{tot}}$ and $\beta^{nr}_{\texttt{vec}}$ of molecules I-VI in the gas phase, cyclohexane, acetonitrile, and water, and the nuclear relaxation contribution to the first hyperpolarizability weight to the total first hyperpolarizability (β^{nr}/β^{el+nr}) .

Molecule	$\beta^{nr}_{\texttt{tot}}$			
	Gas phase	Cyclohexane	Acetonitrile	Water
I	4.32×10^{3}	3.58×10^{4}	4.26×10^{4}	3.80×10^{4}
II	1.67×10^{5}	1.52×10^{6}	3.31×10^{5}	1.57×10^{5}
III	9.02×10^{3}	1.24×10^{4}	2.10×10^{5}	2.59×10^{5}
IV	4.43×10^{6}	5.81×10^{6}	1.30×10^{5}	1.99×10^{5}
V	1.05×10^{4}	3.23×10^{5}	1.02×10^{5}	7.28×10^{4}
VI	1.11×10^{5}	4.99×10^{6}	3.76×10^{5}	2.06×10^{5}
Molecule	$\beta^{nr}_{\texttt{vec}}$			
	Gas phase	Cyclohexane	Acetonitrile	Water
I	-4.32×10^{3}	-1.01×10^{4}	-3.23×10^{4}	-3.02×10^{4}
II	-6.72×10^{4}	-7.96×10^{4}	-7.20×10^{4}	-6.03×10^{4}
III	3.04×10^{3}	-7.09×10^{3}	1.26×10^{5}	2.06×10^{5}
IV	3.04×10^{5}	5.99×10^{4}	-1.03×10^{5}	-9.23×10^{4}
V	1.05×10^{4}	2.16×10^{4}	-5.81×10^{4}	-5.45×10^{4}
VI	-1.00×10^{5}	-1.28×10^{5}	-1.30×10^{5}	-1.18×10^{5}
Molecule	β^{nr}/β^{el+nr}			
	Gas phase	Cyclohexane	Acetonitrile	Water
I	0.79	0.95	0.92	0.91
II	0.99	1.00	0.99	0.99
III	0.93	0.92	0.99	0.99
IV	1.00	1.00	0.88	0.92.
V	0.87	0.99	0.96	0.94
VI	0.99	1.00	0.99	0.98

Table S9: Values of static $\beta^{el}_{\texttt{vec}}/\beta^{el}_{\texttt{total}}$ of investigated molecules in gas phase, cyclohexane, acetonitrile and water at CAM-B3LYP/cc-pVTZ level of theory.

Molecule	$\beta^{el}_{\texttt{vec}}/\beta^{el}_{\texttt{total}}$			
	Gas phase	Cyclohexane	Acetonitrile	Water
I	−1.00	−1.00	−1.00	−1.00
II	−1.00	−1.00	−1.00	−1.00
III	−1.00	−1.00	−1.00	−1.00
IV	−1.00	1.00	−1.00	−1.00
V	1.00	1.00	−1.00	−1.00
VI	−1.00	−1.00	−1.00	−1.00

Table S10: Values of $\beta^{el}_{\texttt{vec}}(-\omega;\omega,0)$ for ω = 1064 nm and $\beta^{nr}_{\texttt{vec}}(-\omega;\omega,0)_{\omega\to\infty}$ of investigated molecules in gas phase, cyclohexane, acetonitrile, and water at CAM-B3LYP/cc-pVTZ level of theory.

Molecule	$\beta^{el}_{\texttt{vec}}(-\omega;\omega,0)$			
	Gas phase	Cyclohexane	Acetonitrile	Water
I	1.35×10^{3}	2.22×10^{3}	2.54×10^{3}	2.54×10^{3}
II	5.95×10^{2}	1.02×10^{3}	9.90×10^{2}	9.79×10^{2}
III	-8.24×10^{2}	1.43×10^{3}	1.50×10^{3}	1.49×10^{3}
IV	-5.32×10^{3}	-1.03×10^{4}	-1.79×10^{4}	-1.83×10^{4}
V	-1.88×10^{3}	-3.07×10^{3}	3.66×10^{3}	3.67×10^{3}
VI	1.38×10^{3}	2.37×10^{3}	2.88×10^{3}	2.90×10^{3}
**Molecule**	**$\beta^{nr}_{\texttt{vec}}(-\omega;\omega,0)_{\omega\to\infty}$**			
	Gas phase	Cyclohexane	Acetonitrile	Water
I	-6.28×10^{2}	-9.92×10^{2}	-2.75×10^{3}	-2.90×10^{3}
II	-2.51×10^{3}	-2.16×10^{3}	-3.64×10^{3}	-3.03×10^{3}
III	2.67×10^{2}	4.80×10^{2}	1.50×10^{3}	2.02×10^{3}
IV	-4.58×10^{3}	-2.42×10^{3}	8.78×10^{3}	9.76×10^{3}
V	1.20×10^{3}	1.46×10^{3}	-3.94×10^{3}	-4.18×10^{3}
VI	-2.98×10^{3}	-3.80×10^{3}	-4.65×10^{3}	-4.64×10^{3}

Table S11: Values of $\beta^{el}_{\mathrm{vec}}(-2\omega;\omega,\omega)$ for ω = 1064 nm of investigated molecules in the gas phase, cyclohexane, acetonitrile, and water at CAM-B3LYP/cc-pVTZ level of theory.

Molecule	$\beta^{el}_{\mathrm{vec}}(-2\omega;\omega,\omega)$			
	Gas phase	Cyclohexane	Acetonitrile	Water
I	2.38×10^3	4.49×10^3	3.89×10^3	3.83×10^3
II	1.18×10^3	2.49×10^3	1.71×10^3	1.64×10^3
III	-1.78×10^3	3.61×10^3	2.73×10^3	2.64×10^3
IV	-1.13×10^4	-2.62×10^4	-4.83×10^4	-4.93×10^4
V	-3.15×10^3	-5.80×10^3	5.36×10^3	5.32×10^3
VI	2.48×10^3	4.86×10^3	4.44×10^3	4.39×10^3

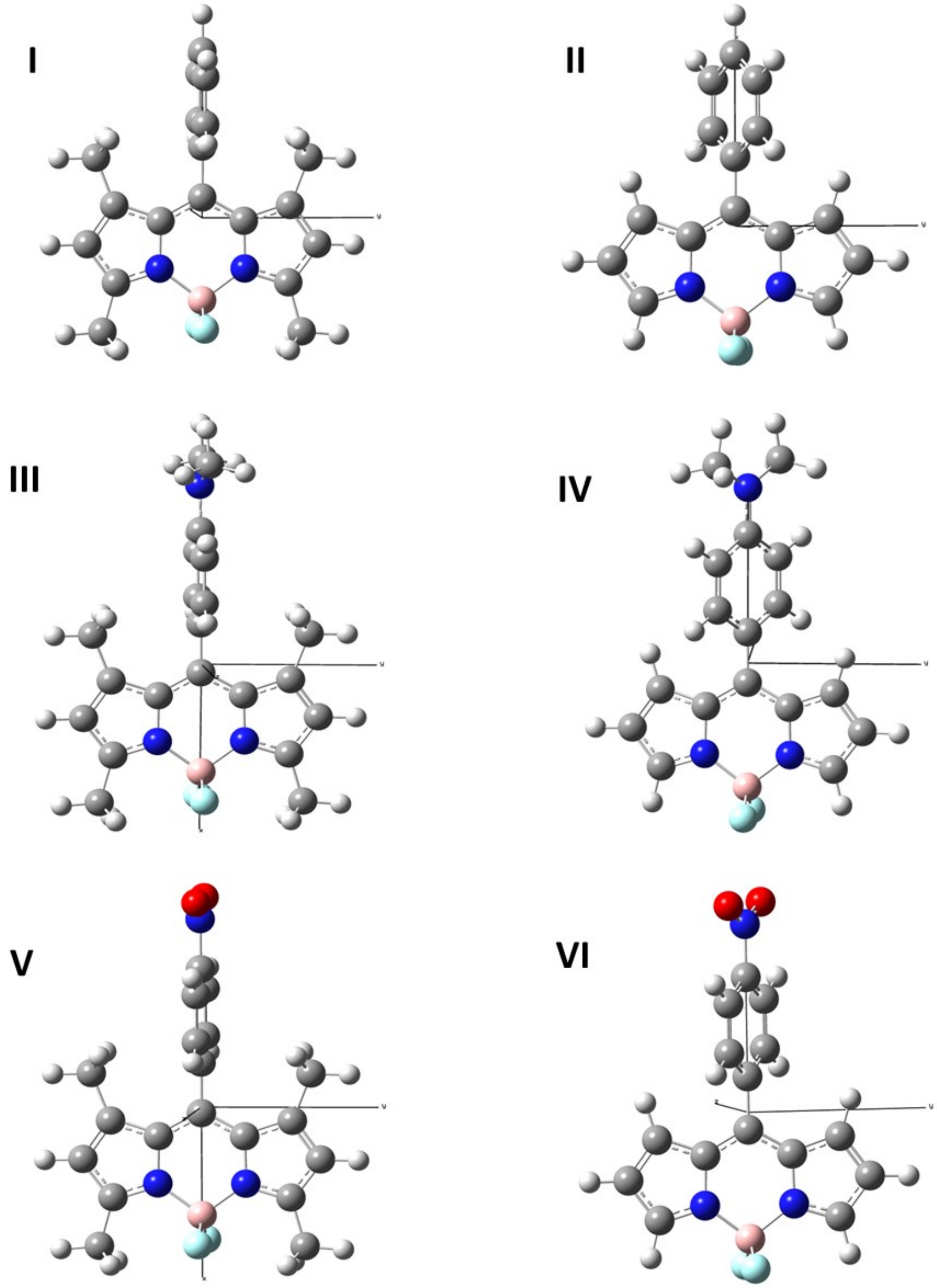

Figure S1: Side views of optimized geometries of Aryl substituted BODIPY molecules I-VI in gas phase at the CAM-B3LYP/cc-pVTZ level of theory.

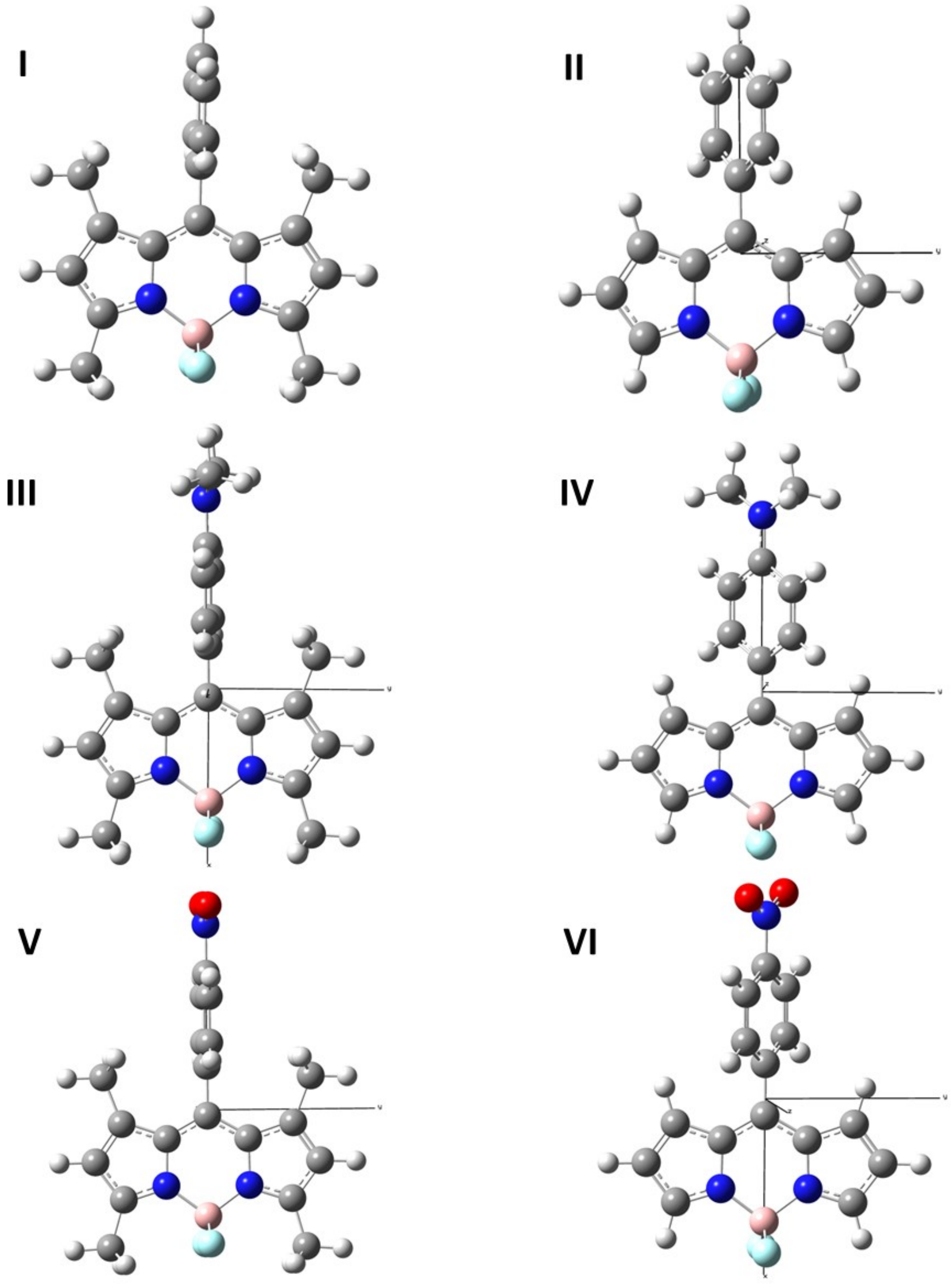

Figure S2: Side views of optimized geometries of Aryl substituted BODIPY molecules I-VI in water at the CAM-B3LYP/cc-pVTZ level of theory.

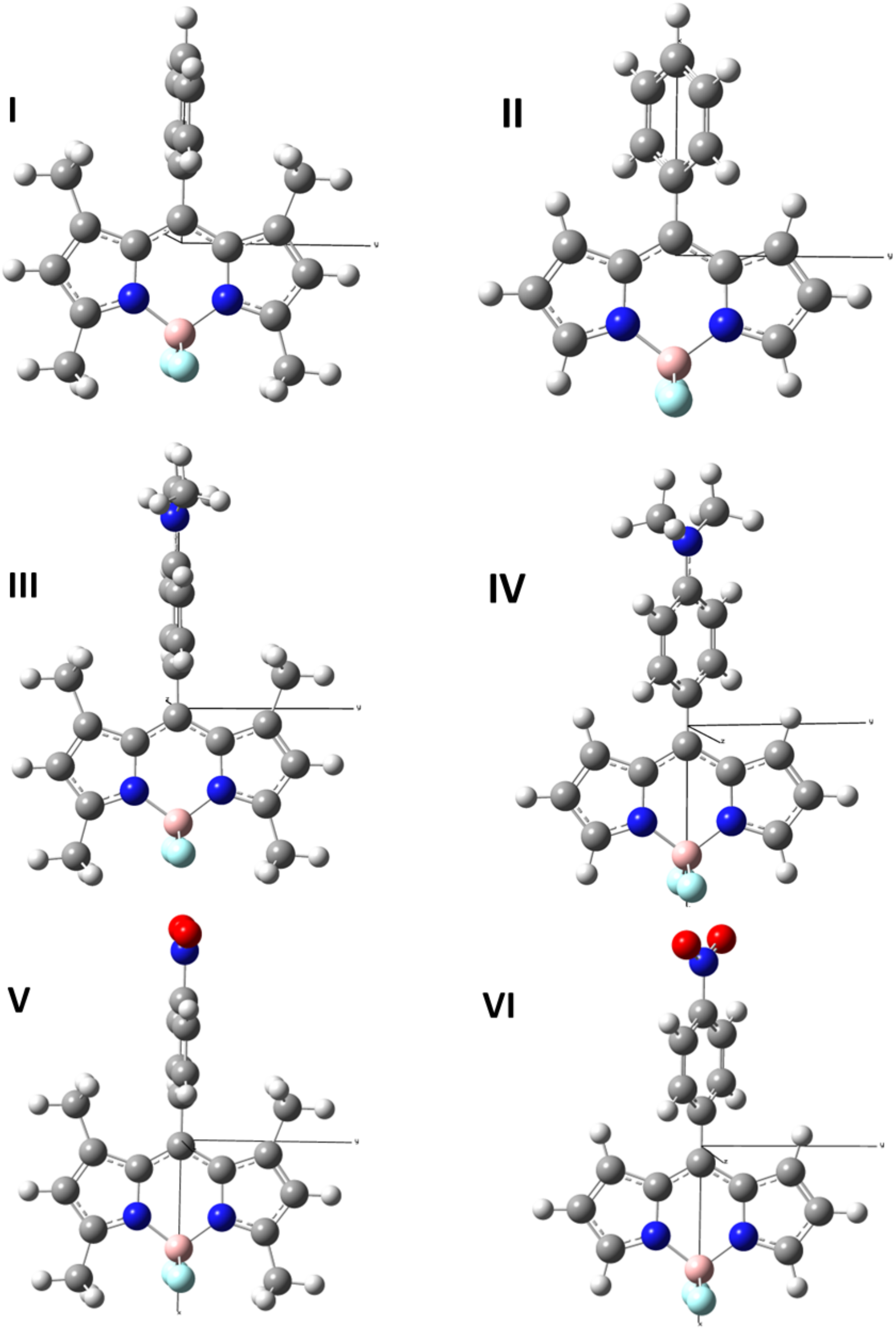

Figure S3: Side views of optimized geometries of Aryl substituted BODIPY molecules I-VI in cyclohexane at the CAM-B3LYP/cc-pVTZ level of theory.

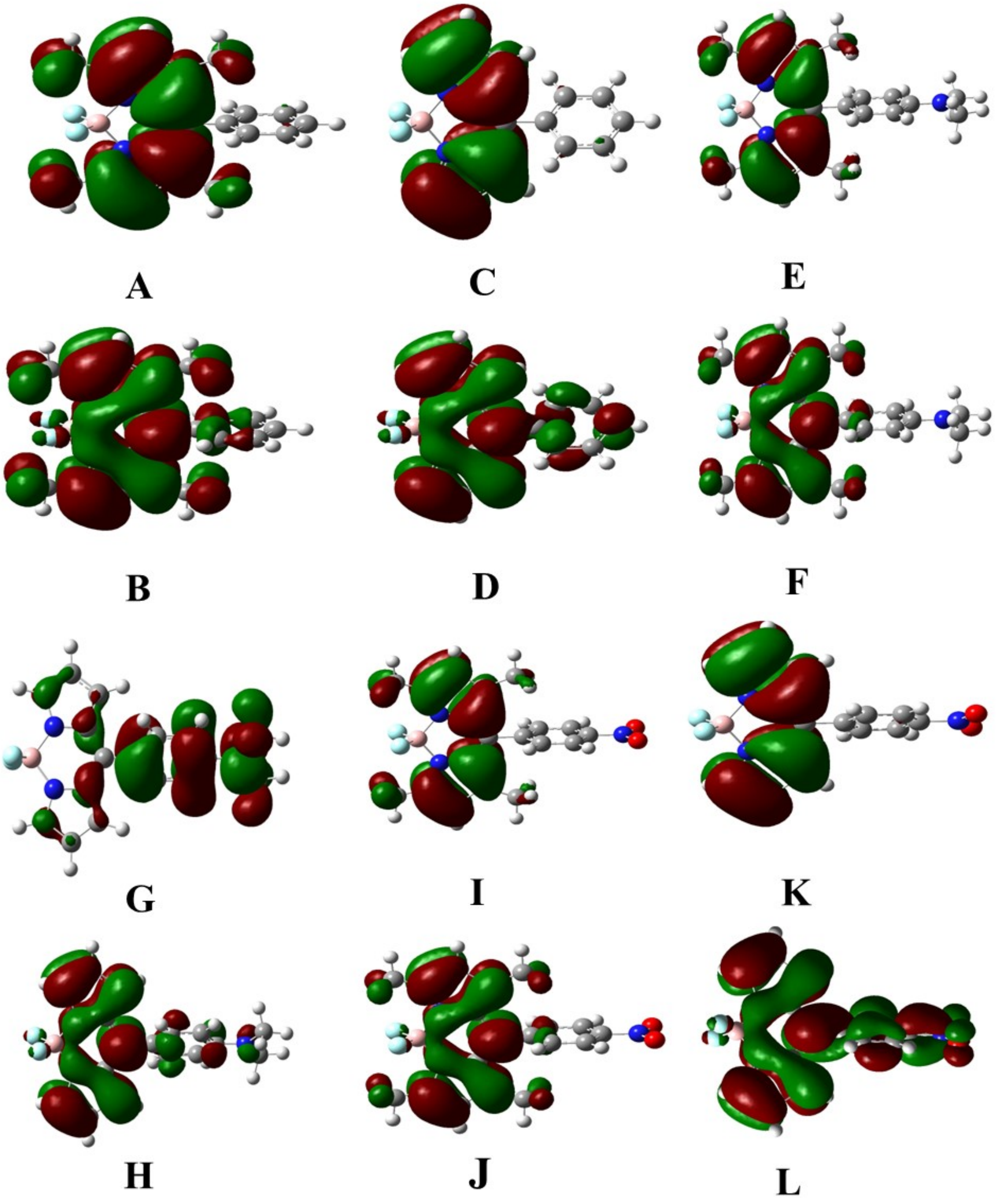

Figure S4: Frontier molecular orbitals of molecule I (A, B), II (C, D), III (E, F), IV(G, H), V (I, J), and VI (K, L) in gas phase at CAM-B3LYP/cc-pVTZ level of theory.

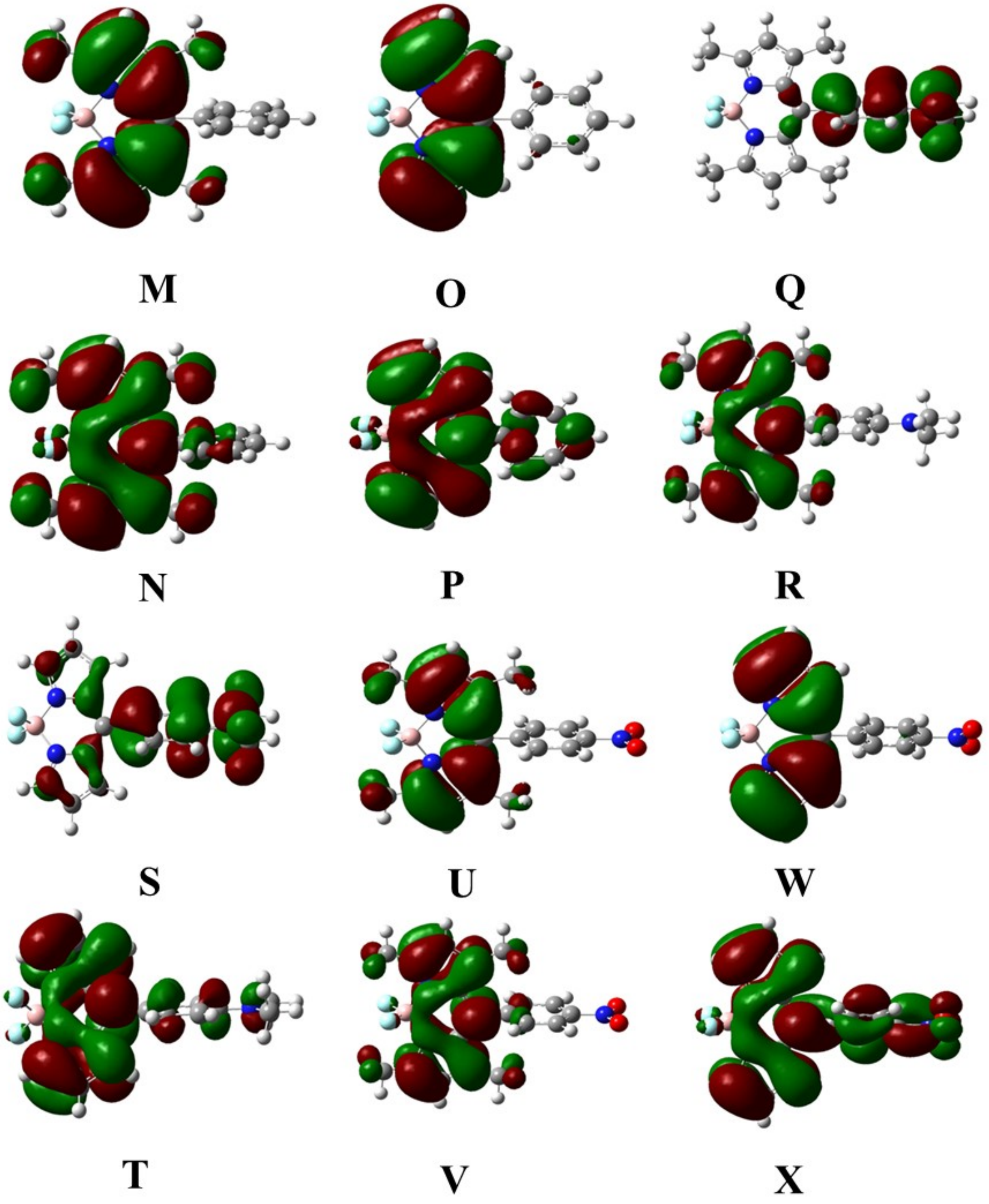

Figure S5: Frontier molecular orbitals of molecule I (M, N), II (O, P), III (Q, R), IV(S, T), V (U, V), and VI (W, X) in water at CAM-B3LYP/cc-pVTZ level of theory.

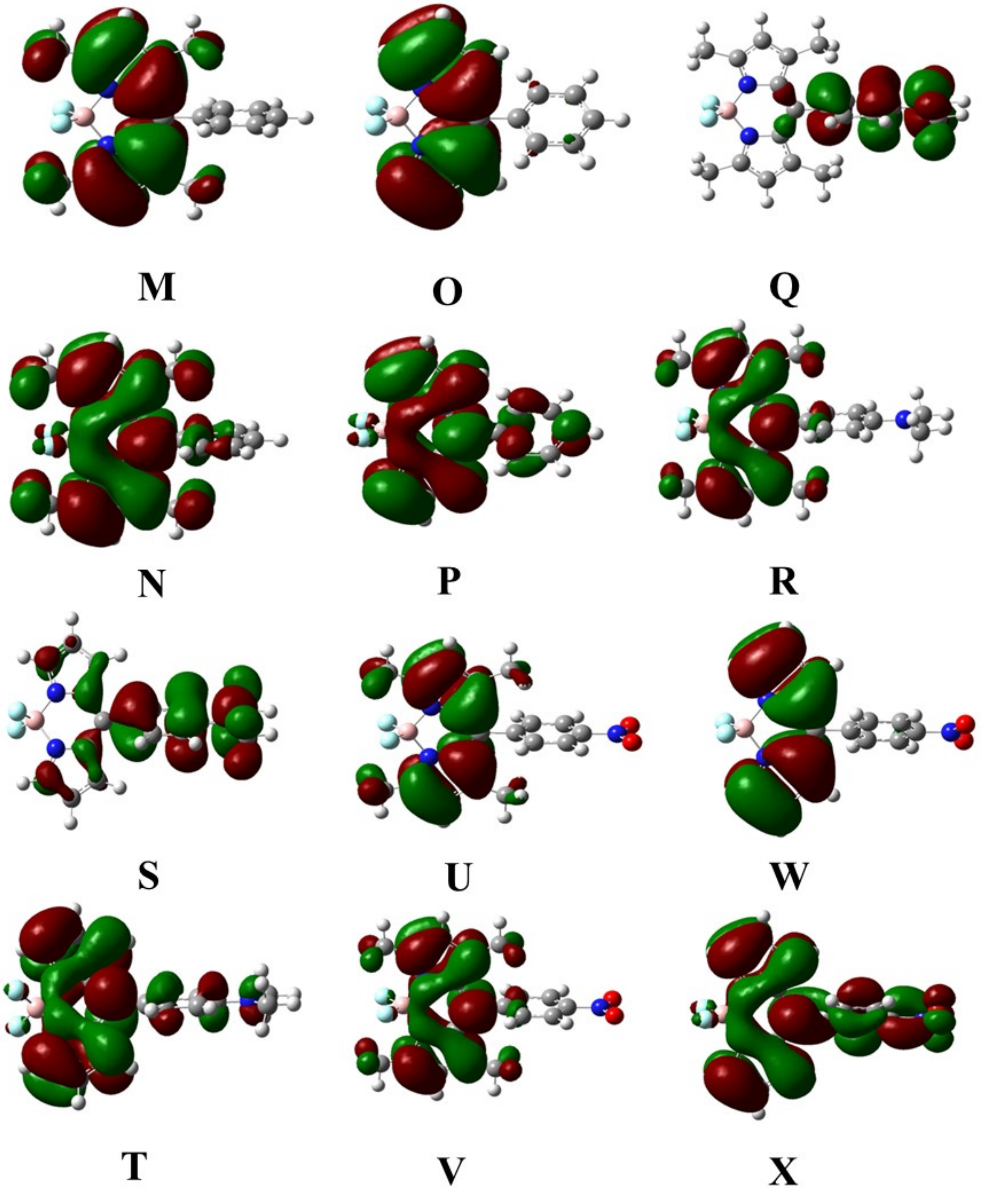

Figure S6: Frontier molecular orbitals of molecule I (M, N), II (O, P), III (Q, R), IV(S, T), V (U, V), and VI (W, X) in cyclohexane at CAM-B3LYP/cc-pVTZ level of theory.

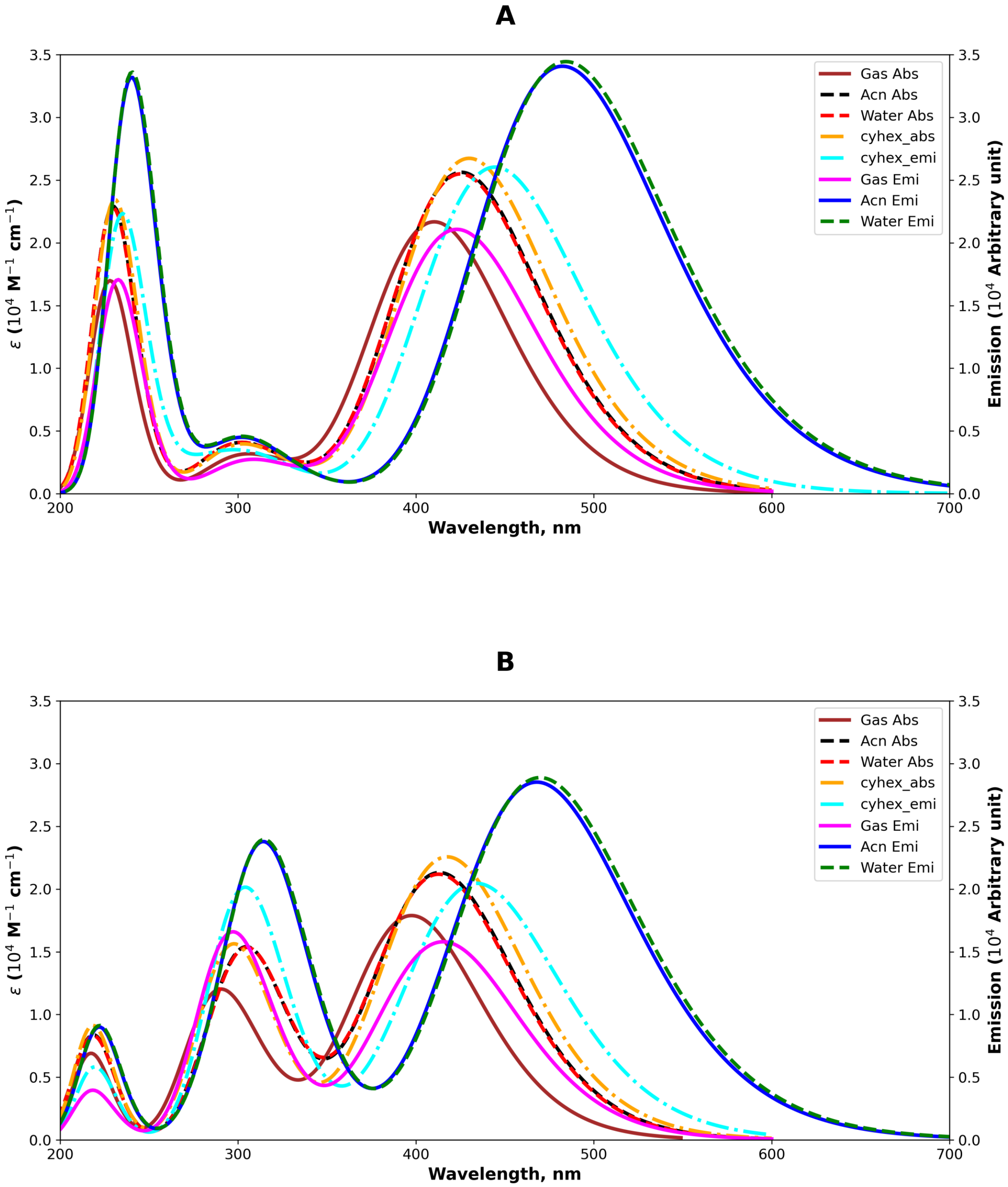

Figure S7: Absorption and emission spectra of molecules I and II.

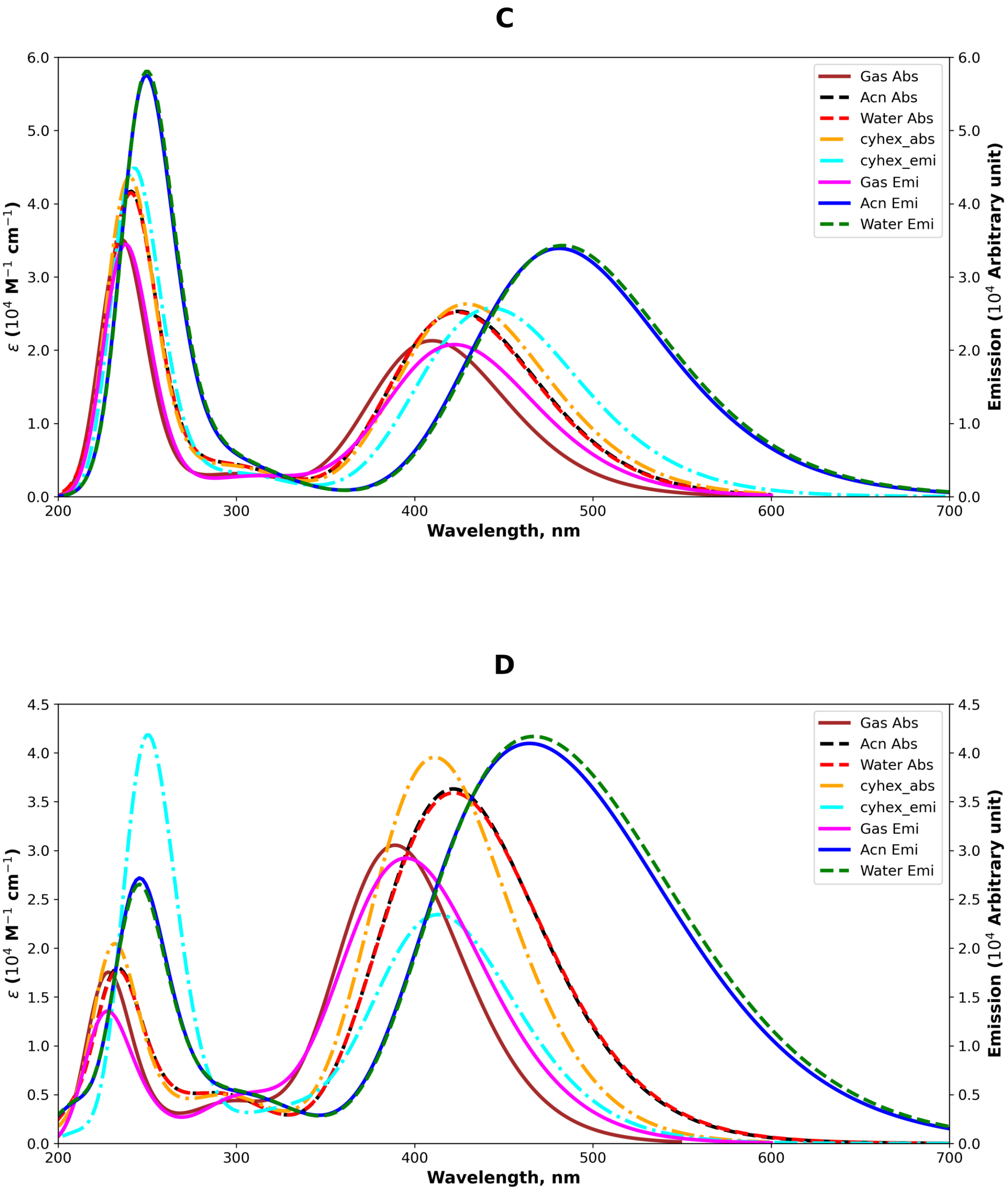

Figure S8: Absorption and emission spectra of molecules III and IV.

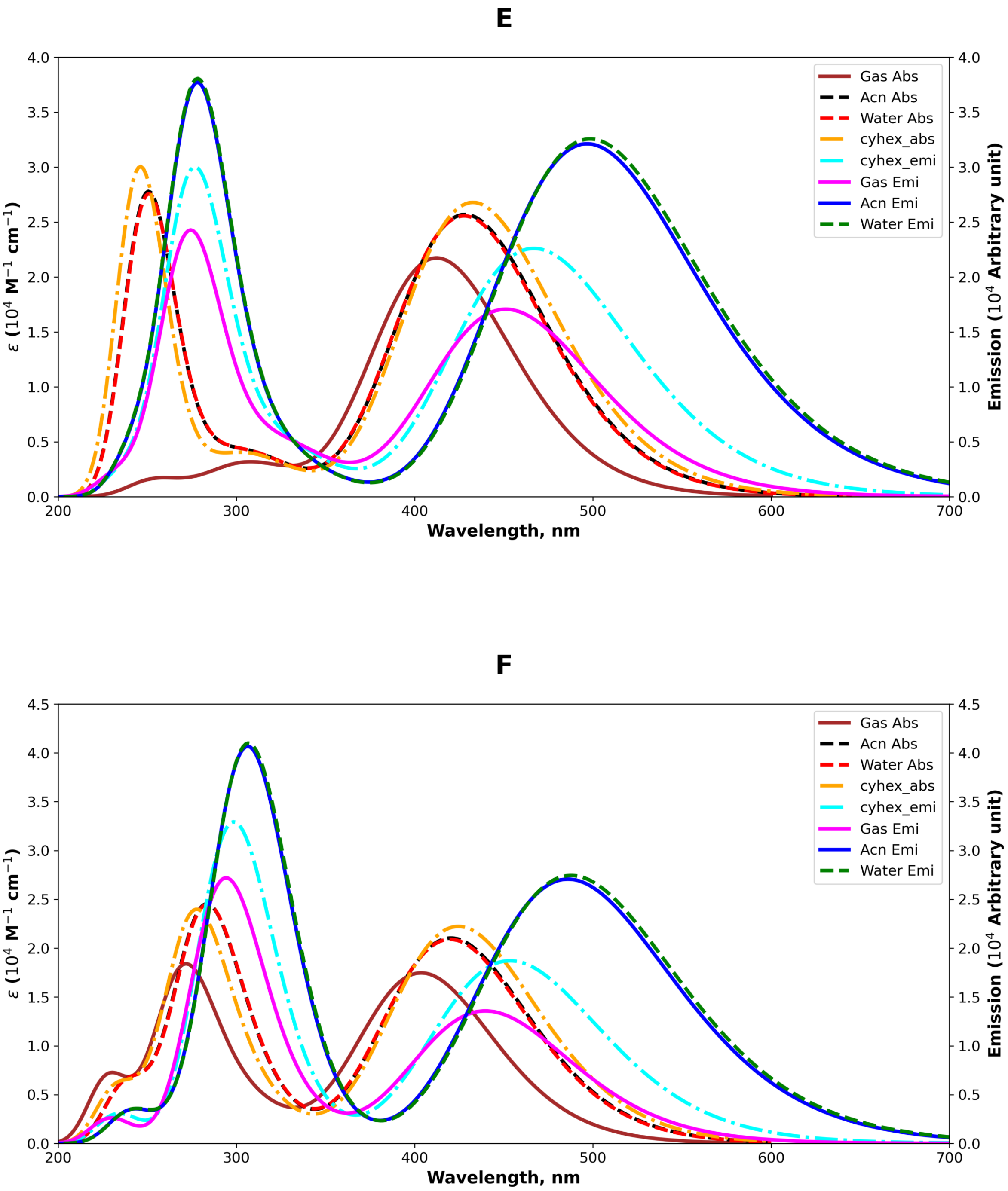

Figure S9: Absorption and emission spectra of molecules V and VI.

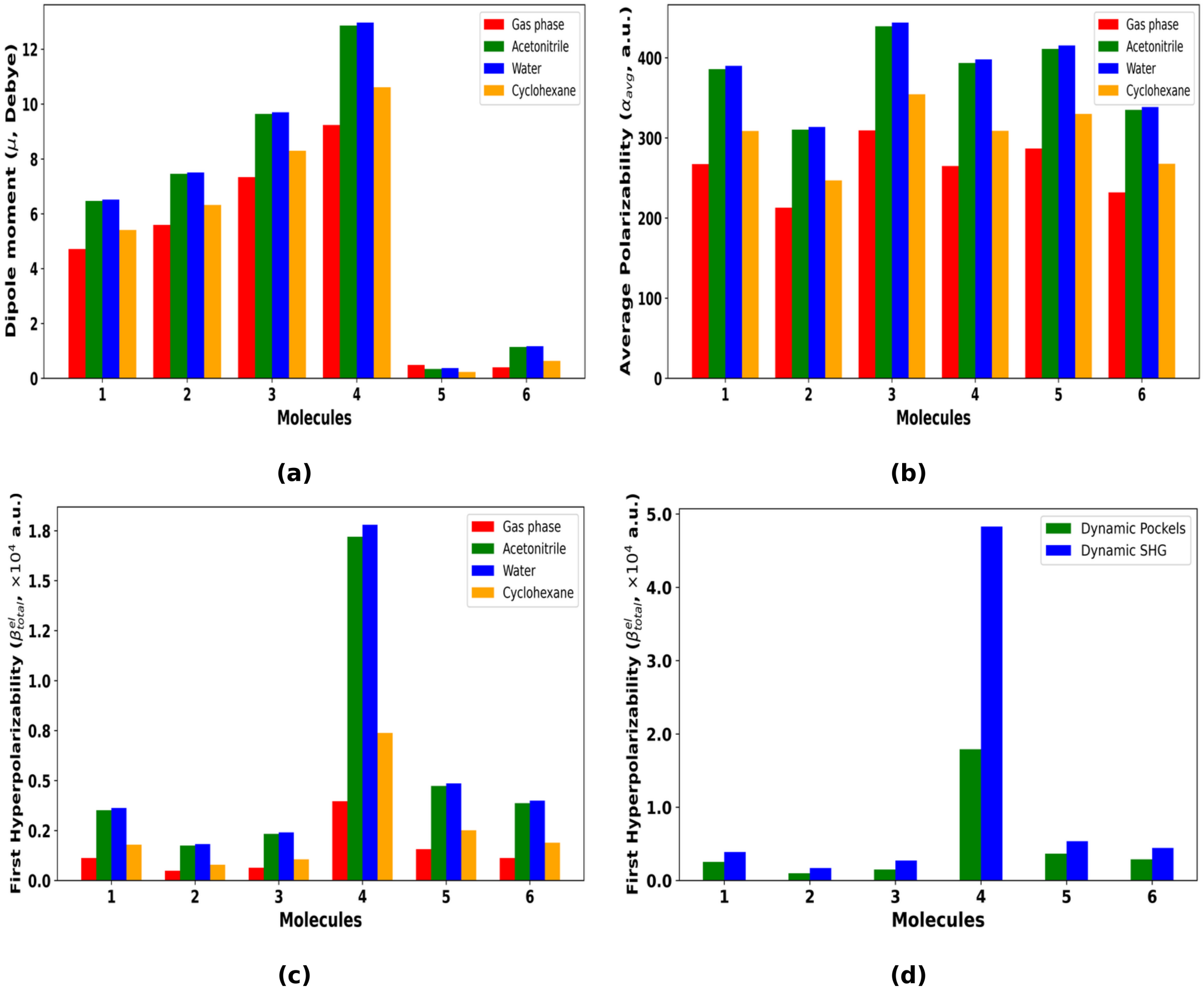

Figure S10: The red, green, blue and orange bars represents (a) dipole moment (μ, Debye), (b) $\alpha_{\mathtt{avg}}$ (a.u), (c) $\beta^{el}_{\mathtt{total}}$ (a.u) in gas phase, cyclohexane, acetonitrile and water respectively and green and blue bars represents Pockels and SHG (d) dynamic first hyperpolarizability in acetonitrile. All calculations of BODIPY dyes were studied using CAM-B3LYP/cc-pVTZ level of theory.

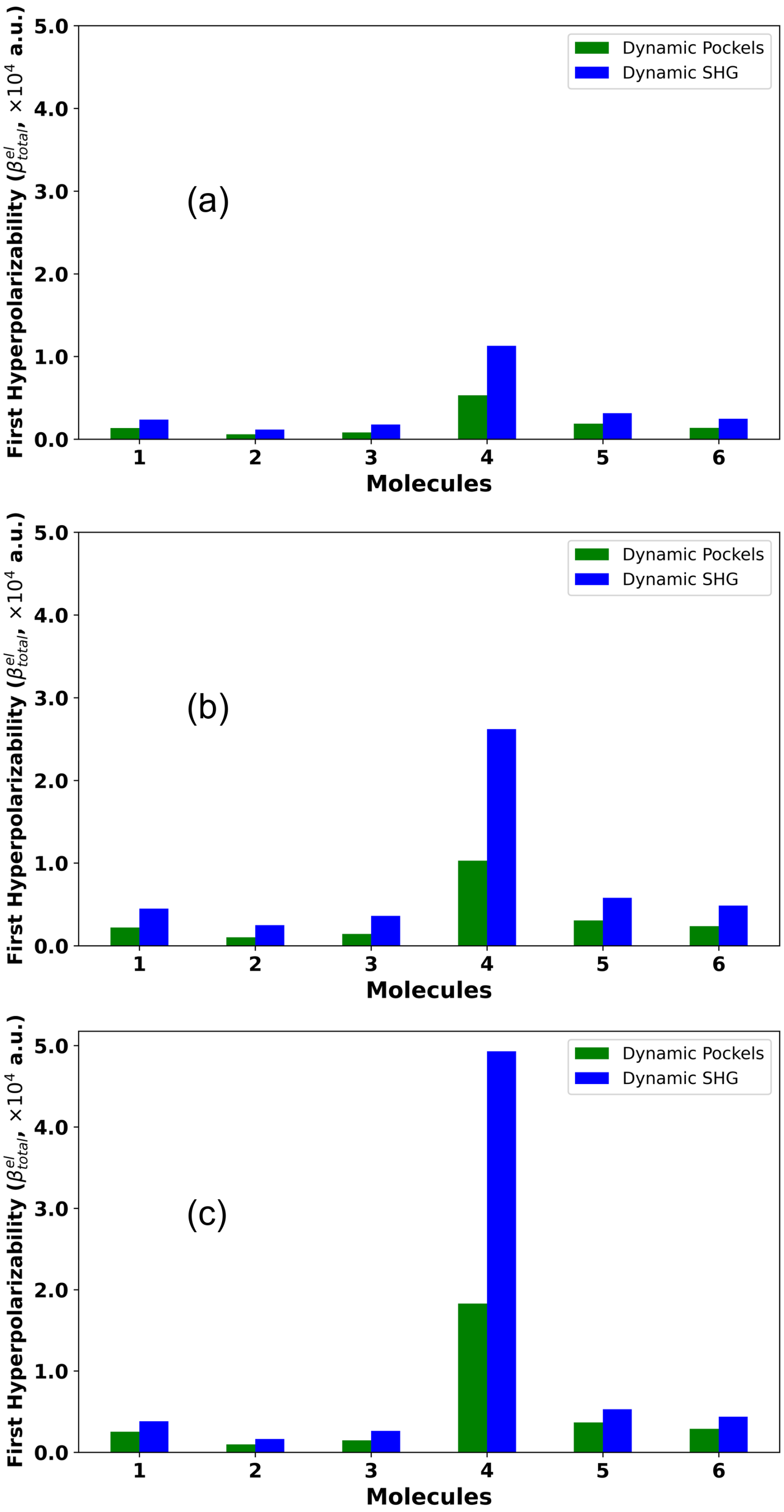

Figure S11: Bar plot of Pockels and SHG values of molecules I-VI in gas phase (a), cyclohexane (b), and water (c).

Optimized structure (CAM-B3LYP / CC-PVTZ) of Bodipy I in gas phase, acetonitrile, water, and cyclohexane:

Table S12: Optimized structure (CAM-B3LYP / CC-PVTZ) for Bodipy I in the gas phase and acetonitrile.

	**Bodipy I in gas phase**			**Bodipy I in acetonitrile**		
C	2.08152800	-0.00010600	-0.00003600	2.08411600	0.00020200	-0.00008200
C	2.78237600	0.00002000	-1.19770700	2.78320700	0.00027000	-1.19902900
C	4.16647900	0.00001100	-1.19737800	4.16771200	0.00028400	-1.19832800
C	4.86164600	-0.00009900	0.00009800	4.86273000	0.00019700	-0.00002400
C	4.16636200	-0.00018800	1.19751400	4.16766200	0.00006500	1.19825500
C	2.78226600	-0.00018600	1.19771200	2.78315900	0.00004600	1.19889800
H	2.23853900	0.00011000	-2.13243600	2.24205600	0.00033300	-2.13518900
H	4.70306100	0.00009300	-2.13596200	4.70361600	0.00036200	-2.13707500
H	4.70285900	-0.00026000	2.13614700	4.70352900	-0.00002800	2.13702300
H	2.23833200	-0.00025300	2.13238400	2.24196700	-0.00006400	2.13503400
C	0.59490900	-0.00006900	-0.00007400	0.59769400	0.00013300	-0.00009600
C	-0.08697600	1.21370100	-0.00012000	-0.08690700	1.21301300	-0.00011000
C	-0.08712100	-1.21376900	-0.00005700	-0.08663100	-1.21288100	-0.00003800
C	0.36974200	2.56136200	-0.00026200	0.36958600	2.55895900	-0.00021200
C	-1.88518800	2.50981300	-0.00020100	-1.88575700	2.51426100	-0.00013400
C	-0.76293600	3.34830700	-0.00026500	-0.76405600	3.34863100	-0.00033300
C	1.76336800	3.09417400	-0.00042300	1.76248200	3.09194300	-0.00033500

Continued on next page

Table S12 – continued from previous page

	Bodipy I in gas phase			Bodipy I in acetonitrile		
H	2.32534700	2.76889400	0.87320800	2.32215300	2.76446200	0.87380300
H	2.32480200	2.76965000	-0.87469700	2.32171700	2.76504500	-0.87498000
H	1.73774900	4.18192900	0.00003800	1.73645300	4.17935700	0.00001600
H	-0.79229300	4.42515700	-0.00034100	-0.79213700	4.42550700	-0.00047400
N	-1.47993800	1.24077500	-0.00011400	-1.48257700	1.23970300	-0.00005700
N	-1.48009100	-1.24066100	-0.00005000	-1.48228400	-1.23992800	0.00004200
B	-2.40716300	0.00011600	0.00040800	-2.39703000	-0.00022400	0.00040900
F	-3.20512900	0.00019100	1.13798300	-3.21332100	-0.00027600	1.13681500
F	-3.20635100	0.00014100	-1.13629500	-3.21439000	-0.00037000	-1.13519700
C	0.36940700	-2.56150600	-0.00019700	0.37023500	-2.55867800	-0.00007300
C	-1.88551500	-2.50964400	-0.00005800	-1.88512200	-2.51459500	-0.00005900
C	-0.76338200	-3.34829100	-0.00016600	-0.76318800	-3.34866400	-0.00015400
H	-0.79289200	-4.42513700	-0.00023500	-0.79096900	-4.42554800	-0.00025100
C	1.76294200	-3.09455700	-0.00036800	1.76330900	-3.09119600	-0.00015400
H	2.32455100	-2.76985100	-0.87445700	2.32255900	-2.76387300	-0.87463200
H	2.32486200	-2.76966500	0.87344700	2.32274200	-2.76374000	0.87415300
H	1.73713000	-4.18230800	-0.00025500	1.73765600	-4.17861800	-0.00007500
C	-3.31771800	-2.90160900	0.00004700	-3.31352400	-2.92150100	-0.00008100
H	-3.82532100	-2.49801600	-0.87504000	-3.82832300	-2.53097700	-0.87681400

Continued on next page

Table S12 – continued from previous page

	Bodipy I in gas phase			Bodipy I in acetonitrile		
H	-3.41208400	-3.98424900	0.00048500	-3.39241100	-4.00491900	0.00036200
H	-3.82538100	-2.49730200	0.87477100	-3.82857600	-2.53022000	0.87616300
C	-3.31733600	2.90198300	-0.00018200	-3.31427000	2.92077700	-0.00011200
H	-3.82492500	2.49853300	-0.87534400	-3.82896000	2.53019500	-0.87688400
H	-3.82513100	2.49768200	0.87446600	-3.82921700	2.52927300	0.87609400
H	-3.41154500	3.98463700	0.00032700	-3.39345300	4.00417300	0.00042800
H	5.94253700	-0.00010000	0.00015200	5.94356600	0.00020300	-0.00000100

Table S13: Optimized structure (CAM-B3LYP / CC-PVTZ) for Bodipy I in water and cyclohexane.

	Bodipy I in water			Bodipy I in cyclohexane		
C	2.08418600	0.00019700	-0.00008100	2.08216900	0.00015300	-0.00007900
C	2.78321800	0.00026600	-1.19907100	2.78249000	0.00020500	-1.19819200
C	4.16773300	0.00028100	-1.19837800	4.16682300	0.00021400	-1.19757600
C	4.86276000	0.00019400	-0.00005200	4.86180000	0.00014800	0.00021000
C	4.16770800	0.00006000	1.19826200	4.16657700	0.00004700	1.19785600
C	2.78319500	0.00004100	1.19892600	2.78224700	0.00003400	1.19818500
H	2.24213400	0.00033100	-2.13526300	2.24003000	0.00025300	-2.13371200
H	4.70361700	0.00036000	-2.13713100	4.70310000	0.00027300	-2.13624100
H	4.70357400	-0.00003200	2.13702500	4.70266100	-0.00002400	2.13663200

Continued on next page

Table S13 – continued from previous page

	Bodipy I in water			Bodipy I in cyclohexane		
H	2.24209000	-0.00006900	2.13510600	2.23958800	-0.00005000	2.13358900
C	0.59776800	0.00013000	-0.00008600	0.59566000	0.00010000	-0.00017100
C	-0.08688800	1.21299800	-0.00009600	-0.08718200	1.21343700	-0.00020600
C	-0.08661800	-1.21286900	-0.00002400	-0.08697400	-1.21333800	-0.00014300
C	0.36959400	2.55890100	-0.00018400	0.36963800	2.56036700	-0.00040400
C	-1.88576500	2.51440200	-0.00011500	-1.88539400	2.51153600	-0.00024900
C	-0.76407200	3.34865300	-0.00030400	-0.76335000	3.34842600	-0.00050300
C	1.76247600	3.09187400	-0.00029600	1.76310700	3.09303600	-0.00060800
H	2.32205400	2.76433000	0.87387700	2.32427800	2.76672900	0.87305800
H	2.32163100	2.76491700	-0.87496900	2.32378600	2.76721200	-0.87477900
H	1.73644700	4.17927500	0.00005700	1.73756100	4.18068900	-0.00031600
H	-0.79211800	4.42552600	-0.00043500	-0.79215400	4.42531300	-0.00068200
N	-1.48263400	1.23968300	-0.00004600	-1.48113200	1.24038900	-0.00013100
N	-1.48234700	-1.23990300	0.00005300	-1.48091100	-1.24055800	-0.00005000
B	-2.39671700	-0.00022000	0.00037300	-2.40350200	-0.00016900	0.00062000
F	-3.21357000	-0.00026900	1.13669200	-3.20837600	-0.00020400	1.13799000
F	-3.21451500	-0.00036300	-1.13523600	-3.21021800	-0.00027900	-1.13542000
C	0.37023000	-2.55862600	-0.00004500	0.37012700	-2.56015500	-0.00028800
C	-1.88514300	-2.51473000	-0.00004000	-1.88491600	-2.51178900	-0.00017700

Continued on next page

Table S13 – continued from previous page

	Bodipy I in water			Bodipy I in cyclohexane		
C	-0.76322200	-3.34868500	-0.00012400	-0.76269600	-3.34845100	-0.00035400
H	-0.79097500	-4.42556500	-0.00021200	-0.79127400	-4.42534400	-0.00050100
C	1.76328500	-3.09114200	-0.00011400	1.76373000	-3.09247400	-0.00046000
H	2.32245800	-2.76376300	-0.87462100	2.32443300	-2.76630400	-0.87448800
H	2.32262800	-2.76362600	0.87422700	2.32471100	-2.76621200	0.87335200
H	1.73762600	-4.17855100	-0.00003300	1.73846700	-4.18013300	-0.00040600
C	-3.31344900	-2.92205600	-0.00007200	-3.31549800	-2.90973800	-0.00009000
H	-3.82846600	-2.53194700	-0.87687200	-3.82581800	-2.51092000	-0.87567100
H	-3.39188600	-4.00549200	0.00037800	-3.40372100	-3.99273700	0.00019800
H	-3.82874000	-2.53117500	0.87621900	-3.82583300	-2.51045500	0.87527400
C	-3.31417900	2.92134700	-0.00010400	-3.31605800	2.90919000	-0.00013200
H	-3.82908800	2.53118400	-0.87694400	-3.82629400	2.51032400	-0.87574200
H	-3.82936800	2.53024300	0.87614800	-3.82631300	2.50974400	0.87520300
H	-3.39290700	4.00476200	0.00044400	-3.40450600	3.99217100	0.00022300
H	5.94359600	0.00020000	-0.00004100	5.94265000	0.00015200	0.00032100

Optimised structure (CAM-B3LYP / CC-PVTZ) of Bodipy II in gas phase, acetonitrile, water and cyclohexane:

Table S14: Optimized structure (CAM-B3LYP / CC-PVTZ) for Bodipy II in gas phase and acetonitrile.

	**Bodipy II in gas phase**			**Bodipy II in acetonitrile**		
C	1.89520900	0.00002300	0.00004300	1.89908400	0.00001800	0.00002600
C	2.60061400	0.69532100	-0.97746600	2.60377000	0.72536100	-0.95723900
C	3.98357400	0.68788900	-0.98015200	3.98689400	0.71714900	-0.96027600
C	4.67804400	-0.00000600	0.00024300	4.68114800	-0.00000300	0.00017200
C	3.98342000	-0.68789100	0.98053600	3.98678300	-0.71714800	0.96054500
C	2.60046000	-0.69529500	0.97764900	2.60365900	-0.72534000	0.95736200
H	2.05959300	1.22518700	-1.74811700	2.06565700	1.27797200	-1.71351500
H	4.51993900	1.22064400	-1.75267600	4.52305600	1.27282200	-1.71633600
H	4.51966200	-1.22066100	1.75313400	4.52285800	-1.27283300	1.71665900
H	2.05931400	-1.22516200	1.74821300	2.06545600	-1.27795200	1.71357400
C	0.41793100	0.00002400	-0.00004700	0.42361600	0.00001800	-0.00004000
C	-0.27765300	1.20619900	0.05794600	-0.27586300	1.20566600	0.05941400
C	-0.27761300	-1.20616800	-0.05819900	-0.27583300	-1.20564300	-0.05959600
C	0.18151600	2.53348500	0.17764700	0.17942700	2.53165300	0.18247900
C	-2.04621000	2.50496000	0.14592100	-2.04846900	2.50649700	0.14904100
C	-0.93026900	3.34657300	0.22588900	-0.93467700	3.34551300	0.23124600
H	-0.95448600	4.41811400	0.31694700	-0.95948700	4.41696400	0.32336000
N	-1.66191600	1.23793700	0.04725300	-1.66238200	1.23519700	0.04752700

Continued on next page

Table S14 – continued from previous page

	Bodipy II in gas phase			Bodipy II in acetonitrile		
N	-1.66187400	-1.23795400	-0.04768100	-1.66235000	-1.23521000	-0.04782900
B	-2.60823800	-0.00005200	0.00039900	-2.59185900	-0.00003700	0.00026200
F	-3.38961300	-0.03916900	1.13600900	-3.39813800	-0.04027100	1.13330800
F	-3.39117900	0.03900200	-1.13412000	-3.39920900	0.04015200	-1.13200200
C	0.18161300	-2.53339400	-0.17834600	0.17950000	-2.53158700	-0.18295600
C	-2.04611800	-2.50495100	-0.14686500	-2.04839900	-2.50649400	-0.14969000
C	-0.93014100	-3.34649900	-0.22703000	-0.93458000	-3.34546200	-0.23202000
H	-0.95431500	-4.41800200	-0.31853700	-0.95935800	-4.41688800	-0.32443800
H	5.75893900	-0.00001600	0.00032100	5.76194900	-0.00001100	0.00022900
H	1.21279100	-2.83385000	-0.23413400	1.20958800	-2.83556200	-0.23826600
H	-3.09271900	-2.76009800	-0.16516500	-3.09348200	-2.76818400	-0.16796000
H	-3.09282100	2.76008200	0.16402300	-3.09355900	2.76816700	0.16717000
H	1.21267800	2.83400400	0.23338600	1.20950300	2.83567400	0.23775400

Table S15: Optimized structure (CAM-B3LYP / CC-PVTZ) for Bodipy II in water and cyclohexane.

	Bodipy II in water			Bodipy II in cyclohexane		
C	1.89919800	0.00001800	0.00002600	1.89656000	0.00085700	0.00154100
C	2.60388100	0.72686500	-0.95615200	2.60549600	0.70292900	-0.96894000
C	3.98700600	0.71865200	-0.95920300	3.98854500	0.69443700	-0.96651700

Continued on next page

Table S15 – continued from previous page

	Bodipy II in water			Bodipy II in cyclohexane		
C	4.68126500	-0.00000200	0.00014900	4.67890100	-0.00054200	0.01220300
C	3.98691100	-0.71864900	0.95943900	3.98042000	-0.69501100	0.98547600
C	2.60378700	-0.72684300	0.95626400	2.59738700	-0.70218400	0.97720800
H	2.06586500	1.28066100	-1.71161400	2.06873100	1.23790600	-1.73894600
H	4.52314900	1.27553000	-1.71437900	4.52804200	1.23203700	-1.73338600
H	4.52298100	-1.27553800	1.71466000	4.51346300	-1.23333500	1.75633700
H	2.06569400	-1.28064100	1.71167100	2.05406400	-1.23707100	1.74268900
C	0.42379300	0.00001700	-0.00003100	0.41990400	0.00105600	-0.00302200
C	-0.27580300	1.20566400	0.05948400	-0.27791800	1.20663200	0.05174200
C	-0.27577400	-1.20564300	-0.05963900	-0.27629600	-1.20511700	-0.06505100
C	0.17934100	2.53162600	0.18277000	0.17890500	2.53454700	0.16231400
C	-2.04855300	2.50652100	0.14907800	-2.04882800	2.50603300	0.12588900
C	-0.93484700	3.34548400	0.23150700	-0.93422400	3.34779700	0.20128600
H	-0.95968200	4.41692600	0.32371700	-0.95943800	4.42012200	0.28251300
N	-1.66238400	1.23511700	0.04743700	-1.66313900	1.23665000	0.03790800
N	-1.66235400	-1.23513100	-0.04771300	-1.66138400	-1.23714600	-0.05735100
B	-2.59144500	-0.00003500	0.00023400	-2.60125700	-0.00217100	0.01842100
F	-3.39840700	-0.04009100	1.13317700	-3.35670200	-0.04351700	1.17775900
F	-3.39936900	0.03998100	-1.13200400	-3.42949300	0.03615900	-1.08930200

Continued on next page

Table S15 – continued from previous page

	Bodipy II in water			Bodipy II in cyclohexane		
C	0.17941000	-2.53156600	-0.18318900	0.18277400	-2.53040800	-0.19604600
C	-2.04848700	-2.50652000	-0.14966900	-2.04516200	-2.50521200	-0.16896600
C	-0.93475600	-3.34543900	-0.23220100	-0.92912800	-3.34415600	-0.25466000
H	-0.95955900	-4.41685700	-0.32468700	-0.95266900	-4.41474500	-0.35662800
H	5.76206200	-0.00000900	0.00019700	5.75976400	-0.00100900	0.01637400
H	1.20944100	-2.83570200	-0.23859300	1.21403200	-2.83041100	-0.25279600
H	-3.09354400	-2.76834300	-0.16790000	-3.09088600	-2.76368500	-0.19101800
H	-3.09361700	2.76832500	0.16717000	-3.09493400	2.76353700	0.14005800
H	1.20936000	2.83580500	0.23815100	1.20951400	2.83725500	0.21594700

Optimised structure (CAM-B3LYP / CC-PVTZ) of Bodipy III in gas phase, acetonitrile, water and cyclohexane:

Table S16: Optimized structure (CAM-B3LYP / CC-PVTZ) for Bodipy III in gas phase and acetonitrile.

	Bodipy III in gas phase			Bodipy III in acetonitrile			
C	-1.22934100	-0.03070600	-0.00073500	C	-1.22682900	-0.03115900	-0.00006400
C	0.25482000	-0.01203800	-0.00008800	C	-1.94272300	-0.03654000	1.18683400
C	0.95811800	-1.21520900	-0.00265300	C	-3.32299500	-0.05180600	1.19669100
C	0.92820800	1.20844000	0.00277100	C	-4.05456000	-0.07133500	-0.00013300
N	2.32366200	1.25327200	0.00032600	C	-3.32294000	-0.05178900	-1.19692000

Continued on next page

Table S16 – continued from previous page

	Bodipy III in gas phase				Bodipy III in acetonitrile		
C	0.45493800	2.54755300	0.01578900	C	-1.94266400	-0.03652400	-1.18699400
N	2.35426600	-1.22562200	0.00017300	H	-1.41094200	-0.02453300	2.12899600
C	0.51803700	-2.56575600	-0.01592000	H	-3.83157400	-0.04840700	2.14702600
C	1.57860500	3.35265400	0.01874100	H	-3.83147300	-0.04837800	-2.14727900
C	-3.32462100	0.00356400	-1.19904800	H	-1.41084400	-0.02450300	-2.12913300
C	-3.32490500	-0.10547700	1.19512200	C	0.25795600	-0.01239000	0.00000000
F	4.07171400	0.03510900	-1.13443700	C	0.95838300	-1.21615300	-0.00013100
B	3.25280200	0.02516800	0.00091600	C	0.92793100	1.20912300	-0.00014400
C	1.66117900	-3.34274900	-0.01883600	C	0.51841200	-2.56880500	-0.00014400
C	-4.05849000	-0.07057600	-0.00286700	C	2.77254500	-2.49029100	0.00013400
C	2.71071300	2.53329400	0.00928400	C	1.66113400	-3.34247400	0.00013400
C	2.77272100	-2.49553900	-0.00904700	H	1.70395900	-4.41896100	0.00032000
C	-1.94458200	0.01984900	-1.18792700	C	0.45384800	2.54969400	-0.00017900
C	-0.94515500	3.06119700	0.02617300	C	2.70910600	2.52911600	0.00010700
C	-1.94473200	-0.09188200	1.18582200	C	1.57640400	3.35243600	0.00009600
F	4.06990500	0.03517400	1.13763900	N	2.35176100	-1.22622900	-0.00030600
C	-6.14962300	0.00303400	1.24392200	N	2.32038600	1.25448500	-0.00032100
N	-5.42907300	-0.11197500	-0.00503400	B	3.26250400	0.02597600	-0.00017900
C	-6.14920400	0.12174500	-1.23757600	F	4.06201200	0.03592800	1.13756700

Continued on next page

Table S16 – continued from previous page

	Bodipy III in gas phase				Bodipy III in acetonitrile		
C	-0.86899100	-3.11340600	-0.02662600	F	4.06306400	0.03592800	-1.13660500
C	4.20625300	-2.88492100	-0.01068600	H	1.59140900	4.42965700	0.00027000
C	4.13424300	2.95789400	0.01040400	C	-0.86895200	-3.11654200	-0.00028300
H	1.59259000	4.42982300	0.02852600	H	-1.43416300	-2.79591000	0.87295900
H	-3.83273400	0.04987800	-2.14842200	H	-1.43403400	-2.79579000	-0.87356800
H	-3.83314100	-0.14405500	2.14480300	H	-0.83208900	-4.20419100	-0.00035200
H	1.70190300	-4.41924100	-0.02876600	C	4.20960400	-2.86486200	0.00058400
H	-1.41521600	0.07629300	-2.12988600	H	4.71256500	-2.45585900	-0.87468600
H	-1.51189100	2.67847700	0.87262700	H	4.71233900	-2.45401100	0.87511600
H	-1.48806000	2.77230500	-0.87201500	H	4.31700000	-3.94636300	0.00167000
H	-0.93466700	4.14759400	0.08297300	C	4.13637700	2.93957100	0.00055700
H	-1.41563500	-0.12409300	2.12905400	H	4.64950200	2.54338600	-0.87471300
H	-7.21373700	-0.07729400	1.04739300	H	4.21657000	4.02343900	0.00162200
H	-5.96540200	0.95740200	1.74847700	H	4.64931300	2.54156800	0.87511100
H	-5.88000300	-0.79885500	1.93158100	C	-0.94723400	3.06236700	-0.00033800
H	-7.21368700	0.02943500	-1.04854000	H	-1.50413600	2.72757100	-0.87364800
H	-5.88412100	-0.61436900	-1.99689100	H	-1.50429200	2.72768800	0.87291400
H	-5.95996600	1.11777600	-1.65154700	H	-0.93743400	4.15061900	-0.00040800
H	-0.83218700	-4.19926500	-0.08278100	C	-6.14708500	0.06335100	-1.24070400

Continued on next page

Table S16 – continued from previous page

	Bodipy III in gas phase				Bodipy III in acetonitrile		
H	-1.44437300	-2.74508000	-0.87364900	C	-6.14714600	0.06333700	1.24034200
H	-1.41923500	-2.83722700	0.87109900	H	-5.88263000	-0.70912200	-1.96389100
H	4.71476100	-2.48982400	-0.88909900	H	-7.21268900	-0.01985000	-1.05020400
H	4.29854200	-3.96732000	-0.00874200	H	-5.95795800	1.03835700	-1.70389900
H	4.71832700	-2.48585800	0.86375200	H	-5.88272500	-0.70914400	1.96353300
H	4.19969900	4.04226400	0.01346600	H	-5.95804200	1.03833900	1.70355600
H	4.65432900	2.57605200	-0.86704100	H	-7.21274000	-0.01986400	1.04978900
H	4.65420400	2.57128200	0.88585900	N	-5.42993700	-0.11286800	-0.00016500

Table S17: Optimized structure (CAM-B3LYP / CC-PVTZ) for Bodipy III in water and cyclohexane.

	Bodipy III in water				Bodipy III in cyclohexane		
C	-1.22941000	-0.03129500	-0.00110600	C	1.22783400	-0.02879900	-0.00037900
C	0.25475100	-0.01242000	-0.00041100	C	1.94349400	-0.01947800	-1.18776900
C	0.95847700	-1.21539200	-0.00307500	C	3.32366600	-0.03472300	-1.19780800
C	0.92781800	1.20822500	0.00277000	C	4.05610200	-0.06851400	-0.00067200
N	2.32333300	1.25350100	0.00046900	C	3.32350500	-0.06347300	1.19683400
C	0.45408900	2.54712700	0.01647400	C	1.94333000	-0.04870700	1.18697800
N	2.35464800	-1.22531500	0.00019300	H	1.41263300	0.00373500	-2.13027400
C	0.51889600	-2.56608300	-0.01716500	H	3.83227200	-0.02029800	-2.14799900

Continued on next page

Table S17 – continued from previous page

	Bodipy III in water				Bodipy III in cyclohexane		
C	1.57758600	3.35264000	0.01976500	H	3.83198900	-0.07113000	2.14716400
C	-3.32484200	0.00589800	-1.19894400	H	1.41232400	-0.04842600	2.12968800
C	-3.32472300	-0.10846700	1.19504300	C	-0.25670800	-0.01111900	-0.00026100
F	4.07189600	0.03599900	-1.13423500	C	-0.95730500	-1.21526800	-0.00113200
B	3.25240600	0.02575200	0.00106000	C	-0.92934600	1.20914200	0.00079400
C	1.66232400	-3.34271600	-0.02013500	C	-0.51571400	-2.56635000	-0.00465100
C	-4.05856600	-0.07078700	-0.00284800	C	-2.77004400	-2.49386200	-0.00251500
C	2.70990400	2.53377800	0.01001100	C	-1.65760000	-3.34270600	-0.00525000
C	2.77360600	-2.49523800	-0.00955200	H	-1.69813000	-4.41927900	-0.00786300
C	-1.94477400	0.02209800	-1.18811300	C	-0.45668600	2.54951900	0.00417700
C	-0.94611200	3.06024300	0.02713100	C	-2.71212900	2.52937600	0.00268100
C	-1.94459400	-0.09508500	1.18548800	C	-1.58037000	3.35216800	0.00504000
F	4.06998900	0.03601200	1.13778500	N	-2.35168400	-1.22693100	-0.00019800
C	-6.14947800	0.00026300	1.24447600	N	-2.32307400	1.25304700	0.00029000
N	-5.42901800	-0.11190300	-0.00486600	B	-3.25889400	0.02369300	0.00029600
C	-6.14938100	0.12418000	-1.23690700	F	-4.06638300	0.03304300	-1.13622100
C	-0.86791400	-3.11421800	-0.02877200	F	-4.06575800	0.03278000	1.13725900
C	4.20715700	-2.88456000	-0.01109200	H	-1.59589500	4.42939200	0.00762100
C	4.13316000	2.95940900	0.01160300	C	0.87236200	-3.11243000	-0.00762700

Continued on next page

Table S17 – continued from previous page

	Bodipy III in water			Bodipy III in cyclohexane			
H	1.59121600	4.42980300	0.03005600	H	1.43954200	-2.77811300	-0.87429200
H	-3.83301300	0.05438900	-2.14820900	H	1.43328900	-2.80204300	0.87203300
H	-3.83285800	-0.14904000	2.14470000	H	0.83669000	-4.19993000	-0.02224100
H	1.70339700	-4.41919100	-0.03065200	C	-4.20534400	-2.87548900	-0.00271900
H	-1.41560800	0.08071200	-2.13003300	H	-4.71177900	-2.46970500	0.87192400
H	-1.51347000	2.67493900	0.87200300	H	-4.71084400	-2.47182000	-0.87891800
H	-1.48809500	2.77366500	-0.87238300	H	-4.30602200	-3.95746200	-0.00156600
H	-0.93615800	4.14647100	0.08688700	C	-4.13825000	2.94429500	0.00334800
H	-1.41530100	-0.12950700	2.12853400	H	-4.65287400	2.55290100	0.87983000
H	-7.21359400	-0.07940300	1.04778900	H	-4.21355100	4.02834400	0.00199300
H	-5.96503300	0.95341700	1.75118600	H	-4.65455500	2.55041000	-0.87098400
H	-5.87990300	-0.80323500	1.93025900	C	0.94367700	3.06344000	0.00661500
H	-7.21381600	0.03169900	-1.04778200	H	1.50362200	2.71550400	0.87264200
H	-5.88448300	-0.61051600	-1.99765200	H	1.49668300	2.74081300	-0.87369100
H	-5.96004300	1.12094400	-1.64900400	H	0.93300400	4.15147100	0.02197700
H	-0.83069100	-4.19992700	-0.08722900	C	6.14801700	0.04345900	1.24272800
H	-1.44351000	-2.74421200	-0.87490900	C	6.14799000	0.07908700	-1.24026400
H	-1.41795300	-2.84013900	0.86972700	H	5.88111100	-0.73992100	1.95295900
H	4.71565800	-2.49044300	-0.88996400	H	7.21298000	-0.03949500	1.05005200

Continued on next page

Table S17 – continued from previous page

	Bodipy III in water				Bodipy III in cyclohexane		
H	4.29935000	-3.96696700	-0.00850500	H	5.96155300	1.01120800	1.72117100
H	4.71946700	-2.48533100	0.86312800	H	5.88527000	-0.68664100	-1.97108000
H	4.19767100	4.04381300	0.01358300	H	5.95711700	1.05813500	-1.69320400
H	4.65426700	2.57724000	-0.86510100	H	7.21322500	-0.00366200	-1.04907000
H	4.65291700	2.57438700	0.88790500	N	5.42921500	-0.10929600	-0.00117800

Optimised structure (CAM-B3LYP / CC-PVTZ) of Bodipy IV in gas phase, acetonitrile, water and cyclohexane:

Table S18: Optimized structure (CAM-B3LYP / CC-PVTZ) for Bodipy IV in gas phase and acetonitrile.

	Bodipy IV in gas phase				Bodipy IV in acetonitrile		
C	0.89761900	-0.00228000	0.00208700	C	0.89992900	0.00016600	-0.00011300
C	1.62264700	-0.83935500	-0.84405400	C	-0.55821900	0.00012400	-0.00029400
C	2.99893700	-0.83880200	-0.85731200	C	-1.27036000	-1.20572300	-0.10244200
C	3.73143500	-0.00572700	0.00500500	C	-1.27057900	1.20587800	0.10140000
C	2.99919100	0.83093500	0.86405400	N	-2.65592100	1.23158300	0.07319100
C	1.62293300	0.83399700	0.84878600	C	-0.82633900	2.52277700	0.30823500
H	1.09361900	-1.48217300	-1.53281600	N	-2.65569300	-1.23171700	-0.07457800
H	3.50858900	-1.48741900	-1.55102800	C	-0.82584300	-2.52228200	-0.31087500
H	3.50904300	1.48060200	1.55665200	C	-1.94669800	3.33126800	0.38207800

Continued on next page

Table S18 – continued from previous page

	Bodipy IV in gas phase			Bodipy IV in acetonitrile			
H	1.09408500	1.47899700	1.53564100	C	3.00261500	-0.88774800	0.81076800
C	5.82392800	-0.87600900	-0.89048300	C	3.00288000	0.88798600	-0.81038900
C	5.82395400	0.88662900	0.87826400	F	-4.39075400	-0.06269900	1.13475700
H	5.61771600	-0.64518400	-1.93927000	B	-3.58238900	-0.00030000	0.00144700
H	6.88896400	-0.75105800	-0.72427300	C	-1.94603100	-3.33089400	-0.38585200
H	5.58058100	-1.92793100	-0.72299300	C	3.73858600	0.00015900	0.00036000
H	5.59207800	1.93461600	0.67061800	C	-3.05244600	2.49578000	0.23866000
H	5.60614800	0.69584900	1.93210200	C	-3.05196400	-2.49577900	-0.24163900
H	6.88922200	0.74453600	0.72814600	C	1.62901500	-0.89024500	0.79372200
N	5.09838200	-0.00918900	0.00832300	C	1.62927500	0.89051700	-0.79376900
C	-0.56906400	-0.00083300	0.00088900	F	-4.39625900	0.06174900	-1.12782200
C	-1.27185000	1.20401600	-0.10585900	C	5.82662900	0.93975500	-0.82428100
C	-1.27428300	-1.20434500	0.10633700	N	5.09597200	0.00019200	0.00068800
C	-0.81809800	2.52213600	-0.30481400	C	5.82619800	-0.94012700	0.82517300
C	-3.04457400	2.49562100	-0.24999700	H	0.19884700	2.82981200	0.40923700
C	-1.93273000	3.33187000	-0.38470900	H	-1.97575700	4.39577600	0.53488700
H	0.21054100	2.82112500	-0.39686900	H	0.19941700	-2.82894100	-0.41229000
H	-4.09214000	2.74534500	-0.27829400	H	3.51245300	-1.57503500	1.46575900
H	-1.95872200	4.39664200	-0.53579100	H	3.51292100	1.57514700	-1.46534600

Continued on next page

Table S18 – continued from previous page

	Bodipy IV in gas phase			Bodipy IV in acetonitrile			
C	-0.82329800	-2.52345600	0.30505100	H	-1.97488100	-4.39519800	-0.54011700
C	-3.04962600	-2.49275100	0.24668000	H	-4.09964300	2.74785400	0.26284600
C	-1.93954600	-3.33116800	0.38254100	H	1.10410800	-1.57236600	1.44637900
H	-1.96775300	-4.39601600	0.53268600	H	-4.09911100	-2.74801200	-0.26630300
H	0.20465100	-2.82439100	0.39846700	H	1.10459700	1.57255800	-1.44669300
H	-4.09769800	-2.74056000	0.27309200	H	6.88988400	0.79865700	-0.66552200
B	-3.60107600	0.00210000	-0.00054600	H	5.58137600	1.97312500	-0.57123800
F	-4.38589400	0.07432300	1.13291300	H	5.62042700	0.79215700	-1.88666200
F	-4.38694400	-0.06853200	-1.13337000	H	6.88955100	-0.79842300	0.66762200
N	-2.65781100	-1.23310600	0.08409900	H	5.58156000	-1.97331800	0.57072400
N	-2.65535100	1.23536900	-0.08585200	H	5.61892700	-0.79381400	1.88750500

Table S19: Optimized structure (CAM-B3LYP / CC-PVTZ) for Bodipy IV in water and cyclohexane.

	Bodipy IV in water			Bodipy IV in cyclohexane			
C	0.89996000	0.00009200	0.00003300	C	-0.89861100	0.00037700	-0.00154300
C	-0.55788000	0.00004900	-0.00008700	C	-1.62603400	0.85632800	-0.82900500
C	-1.27031600	-1.20587300	-0.10198500	C	-3.00141000	0.85396600	-0.84285200
C	-1.27045100	1.20593200	0.10131300	C	-3.73420900	-0.00005200	0.00124700
N	-2.65579000	1.23154000	0.07293800	C	-2.99952100	-0.85414300	0.84360800

Continued on next page

Table S19 – continued from previous page

	Bodipy IV in water				Bodipy IV in cyclohexane		
C	-0.82645800	2.52278200	0.30845300	C	-1.62417800	-0.85606200	0.82706100
N	-2.65565800	-1.23162800	-0.07413100	H	-1.09933200	1.51360500	-1.50566000
C	-0.82617300	-2.52246200	-0.31054200	H	-3.51210300	1.51605600	-1.52283400
C	-1.94699000	3.33125900	0.38227500	H	-3.50867300	-1.51663600	1.52434700
C	3.00279500	-0.88950300	0.80901700	H	-1.09588700	-1.51344300	1.50238100
C	3.00292700	0.88975300	-0.80853100	C	-5.82587000	0.90668300	-0.85597800
F	-4.39141200	-0.06209200	1.13396800	C	-5.82391000	-0.90731300	0.86265000
B	-3.58174600	-0.00020400	0.00109100	H	-5.62439800	0.71763300	-1.91339300
C	-1.94660900	-3.33095800	-0.38544600	H	-6.89000000	0.77684200	-0.68995900
C	3.73876300	0.00009000	0.00026700	H	-5.57817300	1.94971700	-0.64591300
C	-3.05257500	2.49581000	0.23871100	H	-5.57739700	-1.95029100	0.65081700
C	-3.05229700	-2.49579000	-0.24098800	H	-5.61930500	-0.71946300	1.91966000
C	1.62930000	-0.89202100	0.79201100	H	-6.88843800	-0.77668300	0.69985100
C	1.62942900	0.89225300	-0.79176500	N	-5.09747400	-0.00001800	0.00281800
F	-4.39576300	0.06143300	-1.12859600	C	0.56509000	0.00052900	-0.00226300
C	5.82659100	0.94164500	-0.82242500	C	1.27192800	-1.20409500	-0.10811200
N	5.09593800	0.00007800	0.00040300	C	1.27247200	1.20492200	0.10205500
C	5.82637100	-0.94194500	0.82289900	C	0.82193700	-2.52115800	-0.31366000
H	0.19857300	2.83019400	0.40977400	C	3.04826500	-2.49444300	-0.25292300

Continued on next page

Table S19 – continued from previous page

	Bodipy IV in water				Bodipy IV in cyclohexane		
H	-1.97594300	4.39576600	0.53511400	C	1.93881500	-3.33008200	-0.39343700
H	0.19890000	-2.82962100	-0.41220100	H	-0.20553700	-2.82233300	-0.41059700
H	3.51265500	-1.57802500	1.46269300	H	4.09562200	-2.74522400	-0.27988800
H	3.51288700	1.57839000	-1.46200400	H	1.96633200	-4.39426900	-0.54856100
H	-1.97545100	-4.39527800	-0.53960000	C	0.82326200	2.52338900	0.30029400
H	-4.09984800	2.74759600	0.26293200	C	3.04956300	2.49499300	0.24009500
H	1.10455100	-1.57536200	1.44351000	C	1.94058300	3.33211600	0.37543800
H	-4.09954100	-2.74766100	-0.26556900	H	1.96868300	4.39724400	0.52386000
H	1.10479900	1.57563100	-1.44332300	H	-0.20403400	2.82594300	0.39461900
H	6.88984600	0.79944400	-0.66475700	H	4.09705800	2.74530100	0.26627300
H	5.58201600	1.97440600	-0.56631600	B	3.59353900	-0.00110400	0.00737600
H	5.61954400	0.79706000	-1.88501900	F	4.37614700	-0.07157500	1.14905100
H	6.88964500	-0.80041200	0.66475600	F	4.40285300	0.06788800	-1.11531000
H	5.58102200	-1.97454200	0.56688200	N	2.65676400	1.23303900	0.07903500
H	5.61990600	-0.79725100	1.88559600	N	2.65611600	-1.23316900	-0.08494000

Optimised structure (CAM-B3LYP / CC-PVTZ) of Bodipy V in gas phase, acetonitrile, water and cyclohexane:

Table S20: Optimized structure (CAM-B3LYP / CC-PVTZ) for Bodipy V in gas phase and acetonitrile.

	**Bodipy V in gas phase**			**Bodipy V in acetonitrile**			
C	-1.30954600	-0.00000400	-0.00015600	C	1.31257300	-0.00005400	-0.00003200
C	-2.00842800	-0.00001200	-1.20023800	C	2.00858400	0.00003600	-1.20181100
C	-3.38983200	0.00001000	-1.20777300	C	3.38941600	0.00003300	-1.20925200
C	-4.05711100	0.00000800	0.00025200	C	4.05572700	-0.00004000	0.00010700
C	-3.38947600	0.00004400	1.20812600	C	3.38929400	-0.00012000	1.20940400
C	-2.00811900	0.00007300	1.20018200	C	2.00846600	-0.00013200	1.20182200
H	-1.46491300	-0.00008400	-2.13428800	H	1.46842400	0.00010800	-2.13722600
H	-3.95264900	0.00007400	-2.12748400	H	3.94407000	0.00010200	-2.13320000
H	-3.95206000	-0.00000900	2.12798000	H	3.94385800	-0.00017000	2.13340600
H	-1.46428000	0.00018000	2.13404200	H	1.46820800	-0.00019300	2.13718100
C	0.17685900	0.00000800	-0.00025700	C	-0.17412100	-0.00003000	-0.00008100
C	0.85521700	-1.21491200	-0.00036400	C	-0.85462900	1.21393800	-0.00013500
C	0.85525500	1.21489100	-0.00027100	C	-0.85469300	-1.21396600	-0.00007000
C	0.39865000	-2.56388200	-0.00067600	C	-0.39761700	2.56079900	-0.00028900
C	2.65360600	-2.50980600	-0.00051700	C	-2.65287400	2.51422700	-0.00026900
C	1.53098300	-3.34934700	-0.00081800	C	-1.53080200	3.34923700	-0.00036500
C	-0.99420600	-3.09942600	-0.00105700	C	0.99500000	3.09497100	-0.00042000
H	-1.55689400	-2.77894900	0.87407300	H	1.55511300	2.77161000	0.87507700

Continued on next page

Table S20 – continued from previous page

	Bodipy V in gas phase			Bodipy V in acetonitrile			
H	-1.55606800	-2.77964300	-0.87700000	H	1.55467100	2.77218600	-0.87642300
H	-0.96630700	-4.18683100	-0.00064600	H	0.96728900	4.18211900	-0.00006900
H	1.56106400	-4.42608900	-0.00116100	H	-1.55935500	4.42599900	-0.00048300
N	2.24784500	-1.24129700	-0.00033900	N	-2.24992400	1.24010200	-0.00013800
N	2.24786300	1.24134100	-0.00025500	N	-2.24999100	-1.24005000	-0.00006300
B	3.17606400	0.00001300	0.00104400	B	-3.16586200	0.00005100	0.00039300
F	3.97048900	-0.00000400	1.13958300	F	-3.98000800	0.00010700	1.13681500
F	3.97384800	0.00001800	-1.13513200	F	-3.98127400	0.00004000	-1.13508500
C	0.39860000	2.56382900	-0.00059100	C	-0.39776300	-2.56085900	-0.00019300
C	2.65355700	2.50988700	-0.00055300	C	-2.65301700	-2.51415100	-0.00013500
C	1.53088700	3.34936600	-0.00080100	C	-1.53099700	-3.34922800	-0.00022700
H	1.56094700	4.42611300	-0.00110700	H	-1.55961400	-4.42598800	-0.00032200
C	-0.99429400	3.09923300	-0.00077700	C	0.99481500	-3.09512900	-0.00032400
H	-1.55637700	2.77902900	-0.87641700	H	1.55461100	-2.77215900	-0.87617600
H	-1.55668700	2.77905900	0.87466300	H	1.55485100	-2.77203800	0.87532400
H	-0.96650800	4.18664100	-0.00079000	H	0.96702500	-4.18227600	-0.00025100
C	4.08529700	2.90172500	-0.00045600	C	-4.08101700	-2.92087200	-0.00012300
H	4.59254900	2.49750900	-0.87545000	H	-4.59551600	-2.53023600	-0.87697600
H	4.18005700	3.98418300	-0.00020800	H	-4.15984600	-4.00423500	0.00035000

Continued on next page

Table S20 – continued from previous page

	Bodipy V in gas phase				Bodipy V in acetonitrile		
H	4.59250100	2.49710700	0.87438900	H	-4.59573800	-2.52944200	0.87624500
C	4.08535100	-2.90165100	-0.00041500	C	-4.08084900	2.92103400	-0.00031000
H	4.59256900	-2.49758400	-0.87549800	H	-4.59531600	2.53050100	-0.87722900
H	4.59260900	-2.49688900	0.87433300	H	-4.59565000	2.52956400	0.87599200
H	4.18009400	-3.98411000	0.00002500	H	-4.15961400	4.00440200	0.00023900
N	-5.52945100	-0.00000400	0.00047800	N	5.52259400	-0.00000600	0.00018200
O	-6.09031100	0.00062400	-1.07444600	O	6.09328300	-0.00014900	-1.07216800
O	-6.08997600	-0.00067200	1.07557600	O	6.09317300	0.00016000	1.07259000

Table S21: Optimized structure (CAM-B3LYP / CC-PVTZ) for Bodipy V in water and cyclohexane.

	Bodipy V in water				Bodipy V in cyclohexane		
C	1.31266400	-0.00005600	-0.00001800	C	-1.31060400	0.00000100	-0.00010300
C	2.00858400	0.00003400	-1.20184600	C	-2.00807800	-0.00008600	1.20088700
C	3.38939400	0.00003100	-1.20929500	C	-3.38922300	-0.00008700	1.20876600
C	4.05569600	-0.00004200	0.00008500	C	-4.05641600	0.00000300	0.00030900
C	3.38930300	-0.00012200	1.20942000	C	-3.38958200	0.00009200	-1.20834600
C	2.00849700	-0.00013400	1.20186700	C	-2.00843300	0.00008900	-1.20088200
H	1.46852300	0.00010700	-2.13730300	H	-1.46561700	-0.00015300	2.13534700
H	3.94383000	0.00010000	-2.13335700	H	-3.94853600	-0.00015500	2.13034000

Continued on next page

Table S21 – continued from previous page

	Bodipy V in water				Bodipy V in cyclohexane		
H	3.94367300	-0.00017300	2.13352200	H	-3.94917400	0.00016100	-2.12975100
H	1.46836300	-0.00019600	2.13728100	H	-1.46625500	0.00015600	-2.13550700
C	-0.17403600	-0.00003100	-0.00005500	C	0.17592000	0.00000000	-0.00026100
C	-0.85461000	1.21391300	-0.00010900	C	0.85505900	1.21452600	-0.00032200
C	-0.85467600	-1.21394200	-0.00004500	C	0.85505800	-1.21452700	-0.00026000
C	-0.39759200	2.56070800	-0.00024700	C	0.39822800	2.56268300	-0.00047600
C	-2.65286300	2.51435700	-0.00024700	C	2.65328600	2.51157500	-0.00040300
C	-1.53081200	3.34923400	-0.00032400	C	1.53074800	3.34932600	-0.00056300
C	0.99502700	3.09479800	-0.00036000	C	-0.99453400	3.09773100	-0.00061300
H	1.55506400	2.77130700	0.87513100	H	-1.55573800	2.77674100	-0.87643900
H	1.55463900	2.77189600	-0.87635000	H	-1.55602300	2.77650900	0.87493900
H	0.96735500	4.18194000	-0.00000100	H	-0.96671500	4.18504900	-0.00047300
H	-1.55932600	4.42599400	-0.00043000	H	1.56007000	4.42610100	-0.00071500
N	-2.24998100	1.24006300	-0.00012300	N	2.24869500	1.24093100	-0.00030600
N	-2.25005000	-1.24001000	-0.00004800	N	2.24869500	-1.24093200	-0.00021500
B	-3.16553600	0.00005300	0.00035100	B	3.17212200	0.00000000	0.00046900
F	-3.98030300	0.00010800	1.13666100	F	3.97632800	-0.00006300	-1.13582800
F	-3.98142000	0.00004200	-1.13512400	F	3.97441700	0.00006200	1.13815000
C	-0.39774100	-2.56076900	-0.00015100	C	0.39822700	-2.56268400	-0.00039900

Continued on next page

Table S21 – continued from previous page

	Bodipy V in water				Bodipy V in cyclohexane		
C	-2.65300900	-2.51427800	-0.00011300	C	2.65328500	-2.51157600	-0.00032500
C	-1.53101100	-3.34922500	-0.00018600	C	1.53074800	-3.34932700	-0.00047600
H	-1.55959100	-4.42598200	-0.00026900	H	1.56006900	-4.42610100	-0.00060000
C	0.99483900	-3.09496000	-0.00026400	C	-0.99453500	-3.09773100	-0.00051000
H	1.55457700	-2.77187200	-0.87610200	H	-1.55595600	-2.77662500	0.87513000
H	1.55479700	-2.77173900	0.87537900	H	-1.55580700	-2.77662500	-0.87624800
H	0.96708500	-4.18210000	-0.00018300	H	-0.96671500	-4.18504900	-0.00051300
C	-4.08091300	-2.92143200	-0.00012000	C	4.08357200	-2.90902900	-0.00029900
H	-4.59562900	-2.53123000	-0.87705000	H	4.59372800	-2.50838500	0.87454900
H	-4.15927400	-4.00481900	0.00036100	H	4.17248600	-3.99181800	0.00078500
H	-4.59588300	-2.53041800	0.87629800	H	4.59323200	-2.51023000	-0.87628800
C	-4.08074100	2.92159800	-0.00030700	C	4.08357300	2.90902800	-0.00025300
H	-4.59542500	2.53150100	-0.87730300	H	4.59332900	2.50943900	0.87532000
H	-4.59579200	2.53054300	0.87604400	H	4.59363300	2.50917400	-0.87551800
H	-4.15903600	4.00499000	0.00025100	H	4.17248700	3.99181800	-0.00038400
N	5.52240000	-0.00000700	0.00014000	N	-5.52674100	0.00000600	0.00053100
O	6.09333100	-0.00015500	-1.07215700	O	-6.09121900	-0.00013100	1.07450000
O	6.09325000	0.00016900	1.07248100	O	-6.09155000	0.00012200	-1.07326400

Optimised structure (CAM-B3LYP / CC-PVTZ) of Bodipy VI in gas phase, acetonitrile, water and cyclohexane:

Table S22: Optimized structure (CAM-B3LYP / CC-PVTZ) for Bodipy VI in gas phase and acetonitrile.

	Bodipy VI in gas phase			Bodipy VI in acetonitrile			
C	0.99502800	0.00016300	0.00019800	C	-0.99894000	0.00010500	-0.00007700
C	1.69715400	0.68348700	0.98869000	C	-1.69840300	0.69067300	-0.98551200
C	3.07808100	0.68197600	0.99654000	C	-3.07859100	0.68895400	-0.99364600
C	3.74457300	0.00002200	-0.00048000	C	-3.74410400	0.00001000	0.00024400
C	3.07748600	-0.68190200	-0.99719300	C	-3.07831400	-0.68889300	0.99397700
C	1.69661200	-0.68327300	-0.98867800	C	-1.69812700	-0.69052200	0.98551400
H	1.15626800	1.20447000	1.76467300	H	-1.16085400	1.21709900	-1.75953500
H	3.64059500	1.19616800	1.75916900	H	-3.63346100	1.21064200	-1.75600800
H	3.63962500	-1.19615100	-1.76006100	H	-3.63296900	-1.21063900	1.75645500
H	1.15524400	-1.20422200	-1.76434400	H	-1.16035000	-1.21695300	1.75937700
C	-0.48429000	0.00011900	0.00047500	C	0.47974600	0.00009000	-0.00022900
C	-1.17480200	1.20506100	-0.08695500	C	1.17280700	1.20406300	0.08860500
C	-1.17453300	-1.20489600	0.08855900	C	1.17266800	-1.20393400	-0.08942500
C	-0.71373200	2.53069900	-0.23125500	C	0.71396600	2.52794500	0.23345900
C	-2.94173300	2.50165100	-0.20661600	C	2.94235500	2.50349000	0.20961500
C	-1.82420300	3.34190500	-0.30015100	C	1.82613800	3.34047900	0.30385400
H	0.31726200	2.83243400	-0.28749000	H	-0.31678500	2.83098300	0.28694000

Continued on next page

Table S22 – continued from previous page

	Bodipy VI in gas phase				Bodipy VI in acetonitrile		
H	-3.98800500	2.75780600	-0.23127500	H	3.98689000	2.76688900	0.23431100
H	-1.84798700	4.41140700	-0.41200500	H	1.84983800	4.40998400	0.41573400
C	-0.71325600	-2.53015700	0.23576200	C	0.71364000	-2.52759800	-0.23569600
C	-2.94128600	-2.50136700	0.21183900	C	2.94203700	-2.50342500	-0.21232500
H	0.31776500	-2.83168300	0.29277300	H	-0.31716000	-2.83042100	-0.28946400
C	-1.82361200	-3.34130100	0.30712800	C	1.82569800	-3.34018500	-0.30720300
H	-1.84720100	-4.41050600	0.42183900	H	1.84925600	-4.40955500	-0.42040100
H	-3.98751800	-2.75761700	0.23713000	H	3.98653700	-2.76691600	-0.23754200
N	-2.55905000	1.23733000	-0.08137400	N	2.55927700	1.23462400	0.08270600
N	-2.55877600	-1.23737000	0.08337600	N	2.55913200	-1.23465600	-0.08395600
B	-3.50693600	-0.00030700	-0.00266600	B	3.49020000	-0.00020800	0.00126900
F	-4.28111600	-0.07212700	-1.13971700	F	4.29202900	-0.07368000	1.13415200
F	-4.29049600	0.07084300	1.12790100	F	4.29691600	0.07295300	-1.12805900
N	5.21779900	-0.00005100	-0.00086100	N	-5.21205400	-0.00005300	0.00040100
O	5.77765600	0.61400000	0.88158300	O	-5.78152000	0.61417100	-0.87872500
O	5.77714600	-0.61418700	-0.88356800	O	-5.78127800	-0.61422800	0.87972000

Table S23: Optimized structure (CAM-B3LYP / CC-PVTZ) for Bodipy VI in water and cyclohexane.

	**Bodipy VI in water**			**Bodipy VI in cyclohexane**			
C	-0.99906100	0.00009700	-0.00006100	C	-0.99645200	0.00009600	-0.00037800
C	-1.69845600	0.69167900	-0.98484300	C	-1.69754900	0.67843900	-0.99297000
C	-3.07861600	0.68993000	-0.99301800	C	-3.07832100	0.67694100	-1.00067400
C	-3.74411000	0.00000600	0.00022600	C	-3.74436200	-0.00002800	0.00047700
C	-3.07836500	-0.68987800	0.99333000	C	-3.07764500	-0.67697200	1.00119300
C	-1.69820600	-0.69154100	0.98486000	C	-1.69687800	-0.67835600	0.99261700
H	-1.16100300	1.21896200	-1.75832700	H	-1.15810700	1.19503600	-1.77268400
H	-3.63329100	1.21246100	-1.75492500	H	-3.63754900	1.18804700	-1.76742100
H	-3.63284600	-1.21246300	1.75534100	H	-3.63634500	-1.18814400	1.76828000
H	-1.16054700	-1.21882700	1.75820100	H	-1.15688700	-1.19494900	1.77195500
C	0.47959000	0.00008300	-0.00020100	C	0.48281100	0.00012900	-0.00066300
C	1.17274500	1.20403800	0.08867700	C	1.17411700	1.20461300	0.08699900
C	1.17261500	-1.20391900	-0.08942600	C	1.17398000	-1.20438600	-0.08893600
C	0.71398700	2.52785600	0.23371900	C	0.71359800	2.52959300	0.23018900
C	2.94238300	2.50353500	0.20966800	C	2.94177200	2.50254200	0.20723000
C	1.82622100	3.34042700	0.30410900	C	1.82452800	3.34143300	0.29976100
H	-0.31674600	2.83092600	0.28731500	H	-0.31745600	2.83167700	0.28396100
H	3.98688400	2.76709400	0.23431600	H	3.98713800	2.76230900	0.23204700

Continued on next page

Table S23 – continued from previous page

	Bodipy VI in water				Bodipy VI in cyclohexane		
H	1.84990900	4.40992200	0.41608200	H	1.84833200	4.41102400	0.41080100
C	0.71368200	-2.52753200	-0.23580800	C	0.71326400	-2.52901400	-0.23477000
C	2.94208500	-2.50346900	-0.21224500	C	2.94144600	-2.50231000	-0.21193400
H	-0.31709700	-2.83040200	-0.28966000	H	-0.31784400	-2.83078000	-0.28934200
C	1.82581000	-3.34015000	-0.30726500	C	1.82407200	-3.34085700	-0.30611400
H	1.84936500	-4.40951700	-0.42048700	H	1.84773100	-4.41020100	-0.41954900
H	3.98655300	-2.76711200	-0.23741400	H	3.98677500	-2.76218200	-0.23721800
N	2.55927500	1.23454900	0.08264700	N	2.55927200	1.23628100	0.08197400
N	2.55914000	-1.23457800	-0.08383400	N	2.55912300	-1.23625400	-0.08414300
B	3.48976400	-0.00019400	0.00119200	B	3.50043600	-0.00030000	0.00252200
F	4.29240700	-0.07351400	1.13390900	F	4.28480400	-0.07365000	1.13852700
F	4.29700700	0.07283200	-1.12817200	F	4.29417800	0.07254500	-1.12687400
N	-5.21190400	-0.00005100	0.00036800	N	-5.21559400	-0.00006300	0.00095100
O	-5.78161700	0.61374800	-0.87897700	O	-5.77959200	0.61232600	-0.88126600
O	-5.78139900	-0.61379000	0.87989700	O	-5.77899300	-0.61249000	0.88352100

Data Availability

The data that support the findings of this study are available within the article and its supplementary material.
